# Variant‐Specific Landscape of Mutual Exclusivity Among BRAF, EGFR, and KRAS Oncogenes Reveals Overlap With Functionally Antagonistic Mutant Pairs

**DOI:** 10.1002/ijc.70558

**Published:** 2026-06-08

**Authors:** Freya Vaeyens, Jan‐Patrick Hetzel, Khaldoon Abdullah, Carolien Eggermont, Catharina Olsen, Marco Mernberger, Ken Maes, Jelle Vlaeminck, Rainer Claus, Dries Vanisterbecq, Frederik Hes, Martin Pichler, Philippe Giron, Oleg Timofeev, Maxim Noeparast

**Affiliations:** ^1^ Vrije Universiteit Brussel (VUB), Universitair Ziekenhuis Brussel (UZ Brussel) Clinical Sciences, Research Group Genetics, Reproduction and Development (GRAD), Centre for Medical Genetics Brussels Belgium; ^2^ Institute of Molecular Oncology, Member of the German Center for Lung Research (DZL) Philipps University Marburg Germany; ^3^ Institute of Innate Immunity, Biomedical Center (Building 13), 1OG 009, University Hospital University of Bonn Bonn Germany; ^4^ Laboratory of Medical and Molecular Oncology, Oncology Research Center, Faculty of Medicine and Pharmacy Vrije Universiteit Brussel Brussels Belgium; ^5^ Brussels Interuniversity Genomics High Throughput Core (BRIGHTcore), Vrije Universiteit Brussel (VUB) Université Libre de Bruxelles (ULB) Brussels Belgium; ^6^ Interuniversity Institute of Bioinformatics in Brussels (IB2) Université Libre de Bruxelles – Vrije Universiteit Brussel (ULB‐VUB) Brussels Belgium; ^7^ Bavarian Cancer Research Center (BZKF), Augsburg, Germany; Pathology, Faculty of Medicine University of Augsburg Augsburg Germany; ^8^ Vrije Universiteit Brussel (VUB) Universitair Ziekenhuis Brussel (UZ Brussel), Translational Oncology Research Center (TORC), team Hematology and Immunology (HEIM) Brussels Belgium; ^9^ Medical Private University Burgenland (MPUB) Pinkafeld Austria; ^10^ Department of Hematology and Clinical Oncology, University Medical Center and Medical Faculty Augsburg University Augsburg Germany

**Keywords:** cancer genetics, colorectal cancer, functional genomics, lung cancer, mutual exclusivity and functional antagonism

## Abstract

Mutual exclusivity (ME) and co‐occurrence (CO) of oncogenic mutations reflect functional antagonism or dependence and may inform therapeutic strategies. However, most studies overlook variant‐level patterns. We performed a comprehensive, cross‐cohort analysis of BRAF, KRAS, and EGFR mutation subtypes using 64,807 cBioPortal tumor samples, 1570 cancer cell lines, and 2714 Belgian clinical cases. Across all datasets, CO was rare among class I BRAF, hydrolysis KRAS, and classical‐like EGFR mutations. Pairwise variant‐level analyses revealed novel ME interactions, including atypical variants, some overlapping with previously reported synthetically lethal pairs. We functionally validated the ME findings by inducing the expression of EGFR^A289V^ or BRAF^V600E^ in cell lines harboring KRAS^G12D^ or EGFR^exon19del^, respectively, resulting in growth inhibition. Overall survival analysis showed no consistent prognostic disadvantage for CO, except in EGFR–KRAS co‐mutant NSCLC. These findings further refine the ME landscape of BRAF, KRAS, and EGFR variants, offering a variant‐level reference to support mutation‐informed precision oncology.

## What's New?

Mutual exclusivity and co‐occurrence of oncogenic mutations reflect functional antagonism or dependence and may inform therapeutic strategies. However, most studies overlook variant‐level patterns. In this comprehensive, cross‐cohort analysis of BRAF, KRAS, and EGFR mutation subtypes, the most significant mutual exclusivity pairs overlapped with previously explored synthetically lethal pairs. Furthermore, introducing exogenous BRAFV600E or EGFRA289V into cells with endogenous EGFRexon19del or KRASG12D, respectively, led to growth inhibition. Overall survival analysis showed no consistent prognostic disadvantage for co‐occurrence, except in EGFR–KRAS co‐mutant non‐small cell lung cancer. The findings may offer a variant‐level reference to support mutation‐informed precision oncology.

## Graphical abstract

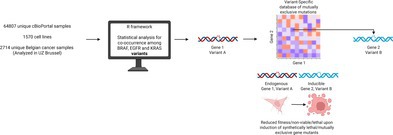

**Abbreviations**
AF: allele frequency
ATP: adenosine triphosphate
BELAC: Belgian accreditation organization
BRAF: v‐rapidly accelerated fibrosarcoma (RAF) murine sarcoma viral oncogene homolog B
CO: co‐occurrence
CRAF: v‐rapidly accelerated fibrosarcoma (RAF) murine leukemia viral oncogene homolog 1
CRC: colorectal cancer
DNA: deoxyribonucleic acid
EGFR: epidermal growth factor receptor
ERK: extracellular signal‐regulated kinase(s)
FFPE: formalin‐fixed paraffin‐embedded
GBM: glioblastoma
GTP: guanosine triphosphate
KRAS: Kirsten rat sarcoma viral oncogene
LUAD: lung adenocarcinoma
MAPK: mitogen‐activated protein kinase
ME: mutual exclusivity
MELA: melanoma
NGS: next‐generation sequencing
NSCLC: non‐small cell lung cancer
OR: odds ratio
OS: overall survival
PACC: P‐loop αC‐helix compressing
PDAC: pancreatic ductal adenocarcinoma
RAS: rat sarcoma viral oncogene
RNA: ribonucleic acid
SE: standard error
SNV: single‐nucleotide variant
TKI: tyrosine kinase inhibitor
WT: wild type

## 1 Introduction

Mutual exclusivity (ME) and co‐occurrence (CO) patterns of gene mutations have drawn long‐standing attention in cancer research and beyond, and they might guide the selection or exclusion of precision oncology treatment choices [1, 2]. These patterns can be associated with epistatic relationships, functional redundancy versus diversification, thereby deepening our understanding of the molecular process of carcinogenesis [1, 3]. The analysis of co‐occurring mutations can shed light on mechanisms underlying tumor invasiveness and therapeutic resistance [4], enabling the discovery of more effective therapies [5]. As analysis of large and complex datasets links genetic findings with clinical outcomes, such patterns are now seen as valuable biomarkers for predicting treatment response and resistance [6, 7, 8].

In recent years, mutually exclusive mutational patterns have contributed to the identification of synthetically lethal targets [1, 9, 10, 11, 12, 13, 14, 15, 16]. Notably, leveraging the vulnerability of cancer cells to the concurrent expression of mutually exclusive KRAS and EGFR mutations has been proposed as a therapeutic strategy [9]. In addition, patterns of mutual exclusivity and co‐occurrence among genetic alterations have also been proposed as a framework for identifying effective immunotherapy‐based treatment combinations [1].

BRAF, KRAS, and EGFR are three frequently mutated genes in cancer that act within the ERK^1^ (extracellular signal‐regulated kinase) pathway. Aberrant activity of the ERK pathway is associated with approximately 40% of human cancers [2, 17]. Mutations in these genes are clinically actionable [18, 19] or under clinical investigation [20]. These mutations have been classified based on their biochemical features and structural impacts, highlighting functional differences that influence signaling pathways and drug responses (see Table S3).

Mutual exclusivity and co‐occurrence patterns among mutations of these oncogenes have been studied across tumor types, though often without considering mutation classes separately [2, 13, 21]. BRAF∩KRAS, and EGFR∩KRAS mutations are generally considered mutually exclusive [1, 14, 22, 23]. Targeted therapies typically exhibit mutation‐specific activity, and different mutations within the same gene can confer distinct pathway dependencies [18, 19, 20, 24, 25, 26, 27, 28, 29, 30]. Small‐molecule compounds targeting specific BRAF, KRAS, and EGFR variants are generally more effective than those targeting the pathway dependencies [31]. Furthermore, mutually exclusive mutational scenarios have been used to explore synthetically lethal targets in ERK‐associated cancer cells [16, 32]. These observations underscore the translational importance of resolving exclusivity and co‐occurrence patterns at the variant level.

In this study, we conducted a cross‐cohort analysis using the publicly available cBioPortal database to characterize mutual exclusivity and co‐occurrence patterns among BRAF, KRAS, and EGFR mutations, with resolution at the level of individual mutations and subtypes. We corroborated our findings using a large dataset of cancer cell lines. Finally, we validated key observations in an independent, manually curated cohort of Belgian cancer patients.

## 2 Materials and Methods

### 2.1 Pilot Analysis

Initially, TSV tables for BRAF‐, KRAS‐, and EGFR‐mutant patient samples and cell lines were extracted from cBioPortal. Class proportions were assessed in Microsoft Excel 2010 to determine the frequencies of mutation types and classes. Fisher's exact test was performed using GraphPad Prism to compare the proportions of mutation classes between the groups.

### 2.2 R Code

The R scripts used to conduct analyses, generate figures, and create tables in this study, along with detailed explanations, are readily accessible to the public (please consult the data availability section). The specific version utilized for this work is R version 4.2.3 (2023‐03‐15 ucrt). The ComplexHeatmap package was utilized to visualize the heatmaps [33, 34].

### 2.3 Source of Data

Patient data on the mutation status of KRAS, BRAF, and EGFR genes from 213 curated, non‐redundant studies were extracted from cBioPortal (Table S1). The dataset was up to date as of November 15, 2022, and included details from 68,479 samples attributed to 64,911 patients. After removing duplicate patients by cross‐referencing patient IDs and excluding multiple samples from the same patient, retaining only the first sample collected, 64,807 patient records remained for further analysis. The frequency of cancer types in the publicly available dataset used for the current study is shown in Figure S1.

For the analysis of mutation status across cell lines, mutation data from 1570 cell lines in the Cancer Cell Line Encyclopedia (Broad Institute, 2019) study, available on cBioPortal, were used. The dataset was current as of December 01, 2022.

### 2.4 Class Assignment

Patients were categorized into mutation classes for KRAS, BRAF, and EGFR genes using established tables derived from the most recent mutational classifications [18, 19, 20]. Single mutations were assigned to their respective mutation classes based on analysis of patient data. Patients with multiple class‐defining mutations were assigned to all corresponding classes, but each class was assigned to each patient only once. Two special considerations applied to the classification of EGFR mutations. First, all deletions spanning amino acids 729–761 were considered exon 19 deletions. Second, some class assignments were based on tuples of mutations rather than single mutations. Therefore, a specific rule was applied: if a patient had a mutation/tuple corresponding to a class assignment and also possessed all mutations of another tuple, the class assignment was based on the tuple with the larger number of mutations. For example, if a patient had mutations L858R and T790M, they were assigned to the tuple “L858R, T790M” (T790M_like_3S) instead of L858R (Classical_like) and T790M (T790M_like_3S) alone. Note that this rule was introduced to assign the sample to the correct class as described in the respective reference.

### 2.5 Statistical Analysis for Co‐Occurrence Between Mutation Classes and Mutated Genes

To analyze the co‐occurrence of mutation classes of gene G0 with the alteration status of gene G1, we constructed a 2 × 2 contingency table for each mutation class of G0 and the alteration status of G1, based on a subset of patients with mutated gene G0. Based on this contingency table with elements A, B, C, and D, we calculated the Odds ratio (OR) according to the following formula: (A × D)/(B × C), the standard error (SE) according to the formula (1/A + 1/B + 1/C + 1/D)^0.5^, and a *p* value using two‐sided Fisher's Exact test.

To tackle 0 values in contingency tables for our calculations, we applied the Haldane correction by adding 0.5 to each value, then calculated the OR and its SE. We set the significance threshold at a *p* value of 0.05. To account for multiple tests on the same dataset and to further report or display significance, a Bonferroni correction was applied.

### 2.6 Statistical Analysis for the Co‐Occurrence of Single Mutations and Altered Genes

To analyze the co‐occurrence of single mutations in gene G0 with the alteration status of gene G1, for each mutation, a 2 × 2 contingency table was created for the presence of G0 mutation and the G1 alteration status, based on a subset of all patients with mutated G0. Using this contingency table, which includes elements A, B, C, and D, we computed the OR, SE, and *p* value, and then conducted significance testing with the Bonferroni correction, following the procedures outlined earlier.

### 2.7 Statistical Analysis of the Co‐Occurrence Between Single Mutations of Two Genes

To examine the co‐occurrence of specific mutations in genes G0 and G1, we used a 2 × 2 contingency table. For each specific mutation pair, we constructed a 2 × 2 contingency table within the defined subset, including patients with either the mutated gene G0 or G1. This table assessed the presence of the G0 and G1 mutations. Within this contingency table, featuring elements A, B, C, and D, we inferred the OR, SE, and *p* value as outlined earlier. The statistical computations followed the methodologies detailed above.

### 2.8 Procedure for Constructing a Curated Belgian Cancer NGS Dataset

In total, 2714 unique patient samples from solid tumors across several tissue types were collected between July 1, 2019, and April 24, 2023. NGS was performed on FFPE‐extracted DNA (Maxwell, Promega) using an in‐house, BELAC‐accredited (ISO15189) capture‐based comprehensive gene panel on the Illumina NovaSeq 6000 to detect somatic variants. To avoid false‐positive co‐occurrences, an in‐house script was used to identify and flag potential sample cross‐contamination. Samples were flagged when > 50% of detected variants (with < 10% allelic frequency) overlapped with high‐frequency variants (> 30% AF) from another sample in the same sequencing run. Only variants from flagged samples with confirmed contamination were removed. Finally, the dataset was limited to the genes BRAF, KRAS, and EGFR, for which true variants were biologically and clinically classified according to the ComPerMed guidelines (https://www.compermed.be [35]). Variants only remained in the dataset if the biological impact was (likely) pathogenic or unknown. The dataset includes 260 BRAF, 636 KRAS, and 184 EGFR variants (SNVs, splice‐site variants, and short indels), of which 63, 37, and 115 are unique gene variants, respectively. The sequencing coverage and quality statistics for each sample are summarized in Table S2.

### 2.9 Kaplan–Meier Method for Assessing Overall Survival Rates in Colorectal Cancer and Non‐Small Cell Lung Cancer Patients' Groups With BRAF, KRAS, or EGFR Mutations

We queried data from 16 non‐redundant colorectal cancer studies involving 6250 patients and 22 non‐redundant non‐small cell lung cancer studies involving 9418 patients (accessible via cBioPortal, 24.01.2024). Data from the patients who harbored BRAF, KRAS, or EGFR mutations were included for further analyses. The data were curated to align with a 60‐month overall survival (OS) analysis. The Kaplan–Meier plots were generated in SPSS (IBM Statistics 24), pairwise comparisons were performed within each group, and significance was reported using the log‐rank (Mantel‐Cox) test and a *p* value of 0.05 or smaller.

### 2.10 Comparative Epistasis Analysis Using CancerEffectSizeR

Somatic mutation data from the Pan‐Cancer Analysis of Whole Genomes (PCAWG) study were retrieved from cBioPortal [36]. The PCAWG cohort comprises 2922 tumor samples, of which 413 harbored at least one somatic mutation in KRAS, EGFR, or BRAF (106 KRAS‐mutant, 273 EGFR‐mutant, and 49 BRAF‐mutant samples), retaining a single sample per patient. Variant‐level epistasis was independently assessed using CancerEffectSizeR [37] in parallel to Fisher's exact test–based pipeline described above. CancerEffectSizeR analyses were performed in R (version 4.2.3) using the hg19 reference genome and the *ces.refset.hg19* reference set. Whole‐genome sequencing data were loaded with the specified genome‐wide coverage. Trinucleotide mutation rates were estimated using COSMIC mutational signatures (v3.2). No tissue‐specific mutational signature constraints were applied, as cancer entity nomenclature used in the PCAWG study could not be unambiguously mapped to the tissue classification scheme implemented in the CancerEffectSizeR package. Gene‐specific mutation rates were calculated using the default covariates implemented in CancerEffectSizeR. Variant‐level recurrence was assessed prior to epistasis testing. Epistatic interactions were subsequently computed for all pairwise combinations of variants in EGFR–KRAS, EGFR–BRAF, and BRAF–KRAS, using a confidence level of 95%, and the resulting variant‐pair *p* values were used for downstream comparison with the Fisher's exact test–based analysis.

### 2.11 Cell Culture and Lentiviral Infection

The intestinal adenocarcinoma cell line LS513 (RRID: CVCL_1386), which harbors the KRAS^G12D^ mutation, was purchased from Addexbio. The lung adenocarcinoma cell line PC‐9 (RRID: CVCL_B260), which harbors EGFR^E746‐A750del^ and TP53^R248Q^ mutations and was originally obtained from ATCC, was available in‐house. All cell lines were authenticated using short tandem repeat (STR) profiling within the last three years. Cells were maintained on standard tissue culture plates (Greiner) in RPMI medium supplemented with 100 U/mL Penicillin–Streptomycin (both from Gibco) and 10% FBS (Merck) at ambient oxygen in a humidified cell culture incubator (37°C with 5% CO2). Lentivirus particles containing doxycycline‐inducible constructs expressing BRAF (wild‐type or V600E mutant), EGFR (wild‐type or A289V mutant) coupled with GFP via P2A, or GFP alone (control) were produced by Thermo Fisher Scientific. The constructs were cloned into pCW57‐MCS1‐2A‐MCS2 (Addgene #71782). For lentiviral infection, cells were plated in 6‐well plates at 1 × 10^5^ cells/well, and the next day, spin infection with 2 × 10^5^ PFU was done (1 round, 60 min at 500 g) in antibiotic‐free medium containing 6 μg/mL polybrene (Sigma). 24 h after infection, the media were changed, and puromycin selection (Invivogen) was initiated. Transduced PC‐9 and LS513 cells were selected with 0.6 and 0.8 μg/mL puromycin for 5 and 12 days, respectively. Puromycin‐resistant polyclonal cells were used in all experiments, transgene expression was induced with 1 μg/mL doxycycline (Sigma), and media were refreshed every 48 h. Cell viability was measured using CellTiter‐Glo Luminescent Cell Viability Assay (Promega) according to the manufacturer's instructions. All experiments were performed with mycoplasma‐free cells.

### 2.12 Lentiviral Vector Construction and Production

Lentiviral production was done by Thermo Fisher Scientific. For the lentiviral vector construction, each gene of interest and EmGFP were cloned into the third generation of all‐in‐one doxycycline inducible lentiviral vector, pCW57‐MCS1‐2A‐MCS2 (Addgene plasmid #71782). As a negative control, the stuffer sequence and EmGFP were cloned into the same plasmid with the same strategy, and then the prepared transfer plasmids were used for lentiviral vector production.

For lentiviral vector production, Gibco LV‐MAX Production System including Viral Production Cells, LV‐MAX Production Medium, and LV‐MAX Transfection kit (Thermo Fisher Scientific, Cat. No. A35684), and LV‐MAX Lentiviral Packaging Mix (Thermo Fisher Scientific, Cat. No. A43237) were used. Briefly, Viral Production Cells were transfected with each prepared plasmid and LV‐MAX Lentiviral Packaging Mix in a 2:3 ratio in Gibco Opti‐MEM I Reduced Serum Medium (Thermo Fisher Scientific, Cat. No. 31985062) with LV‐MAX transfection reagent. The transfected cells were incubated at 37°C for 48 h in a humidified atmosphere of 8% CO2 in air on an orbital shaker at 125 rpm (19 mm shaking diameter). The viral supernatant was collected by centrifugation at 1300 × g for 15 min at 48 h post‐transfection. The collected viral particles were concentrated with 20% (w/v) of Polyethylene glycol 6000 (Merck KGaA, Cat. No. 8.07491.5000), and the final concentrated viral particles were resuspended in Gibco DPBS (Thermo Fisher Scientific, Cat. No. 14190144), followed by QC for titer determination via p24 ELISA assay.

### 2.13 Live Cell Imaging

For quantitative real‐time monitoring of cell confluency, the automated time‐lapse microscope IncuCyte S3 (Sartorius) with S3/SX1 G/R optical module was used. Prior to imaging, cells were seeded in triplicate at densities of 4000 and 8000 cells/well for PC‐9 and LS513, respectively. Treatment with doxycycline began 24 h after plating. GFP fluorescence and phase‐contrast images were taken every 12 h for up to 9 days. Confluence and the GFP intensity (indicative of the number of GFP‐positive cells) per well were calculated using the IncuCyte Zoom 2022 software.

### 2.14 PCR, Reverse‐Transcription Real‐Time PCR (RT‐qPCR)

The integration and mRNA expression of transgenes were confirmed by PCR and RT‐qPCR, respectively, using cDNA‐specific primers: 5′ GAGAACTGCCAGAAACTGACCAA and 5′ TCTCATAGCTGTCGGCCCCA (EGFR), 5′ ACAGTGGGACAAAGAATTGGATCTG and 5′ TCTGGTGCCATCCACAAAATGGA (BRAF), and 5′ GACCACATGAAGCAGCACGAC and 5′ GCTTGTCGGCGGTGATATAGAC (emGFP). We used these same EGFR and BRAF primers for Sanger sequencing of the PCR amplicons to confirm the presence of either the wild‐type or the mutated transgene. We isolated genomic DNA from the cells with the Qiagen DNeasy Blood & Tissue Kit. We used 50 ng of DNA for the PCR reaction to detect transgene integration. For RT‐PCR, total RNA was isolated with the RNeasy Mini kit (Qiagen). The cDNA was generated with the SuperScript VILO cDNA Synthesis Kit (Invitrogen). Gene expression in non‐induced and induced samples collected 48 h after the start of doxycycline treatment was analyzed on a LightCycler 480 (Roche) using SYBR Green (Thermo Fisher Scientific) with the gene‐specific primers mentioned above. For normalization of mRNA expression levels, the GAPDH gene expression levels served as control (forward: 5′ GCTCATTTCCTGGTATGACAACG and reverse: 5′ GAGATTCAGTGTGGTGGGGG).

## 3 Results

### 3.1 Discovery of BRAF, KRAS, and EGFR Mutation Types With Mutually Exclusive or Co‐Occurring Tendencies

To explore how patterns of mutual exclusivity and co‐occurrence manifest at the level of specific BRAF, KRAS, and EGFR mutations, we first categorized each variant into functional classes based on previously established mechanistic criteria [18, 19, 20] (Table S3). Our analysis focused exclusively on small nucleotide variants, indels, and splice variants, excluding structural variants and copy number alterations.

**FIGURE 1:**
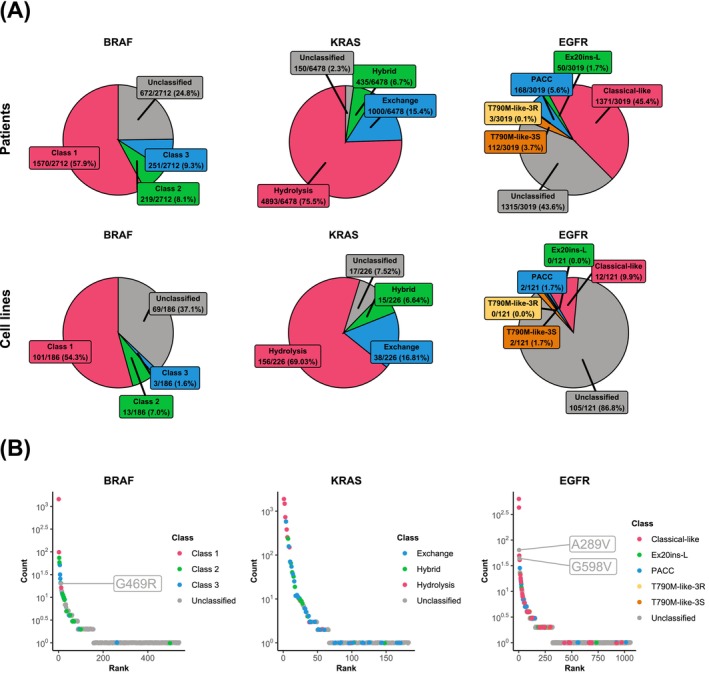
Frequency of mutational classes and mutational types in human cancer. (A) Absolute and relative frequency of BRAF, KRAS, and EGFR mutational classes in human cancer (as described in Table S3) among patient samples (top) and cell lines (bottom). Different classes of each gene are color‐coded and will appear accordingly in the following figures. We queried data of 68,479 samples from 64,911 cancer patients, encompassing 2710, 6442, and 2999 samples with BRAF, KRAS, and EGFR mutations, respectively. Note that total mutation counts and class‐assigned mutation counts, as displayed in (A), are higher than the sample counts because some samples harbored more than one mutation of the same gene, which had to be acknowledged when determining the relative frequency of each variant. Duplicate patient data were removed as described in the methods section, and concerning multiple samples of the same patient, only the earliest sample was considered. For cell lines, 1570 cell lines in the Cancer Cell Line Encyclopedia were queried, and those with BRAF, KRAS, or EGFR mutations were considered for this analysis. (B) Scatter plots show the absolute count of each variant against its frequency rank for BRAF (left), KRAS (middle), and EGFR (right) mutations, with variants assigned to their respective classes. Lower ranks indicate a higher prevalence in human cancer.

We then assessed the frequency distribution of mutation classes across large public cancer cohorts and cell lines (Figure 1A) and determined the most recurrent variants within each gene (Figure 1B). Notably, several atypical but therapeutically relevant variants, including BRAF^G469R^, EGFR^A289V^, and EGFR^G598V^, appeared among the top ten [38, 39, 40].

**FIGURE 2:**
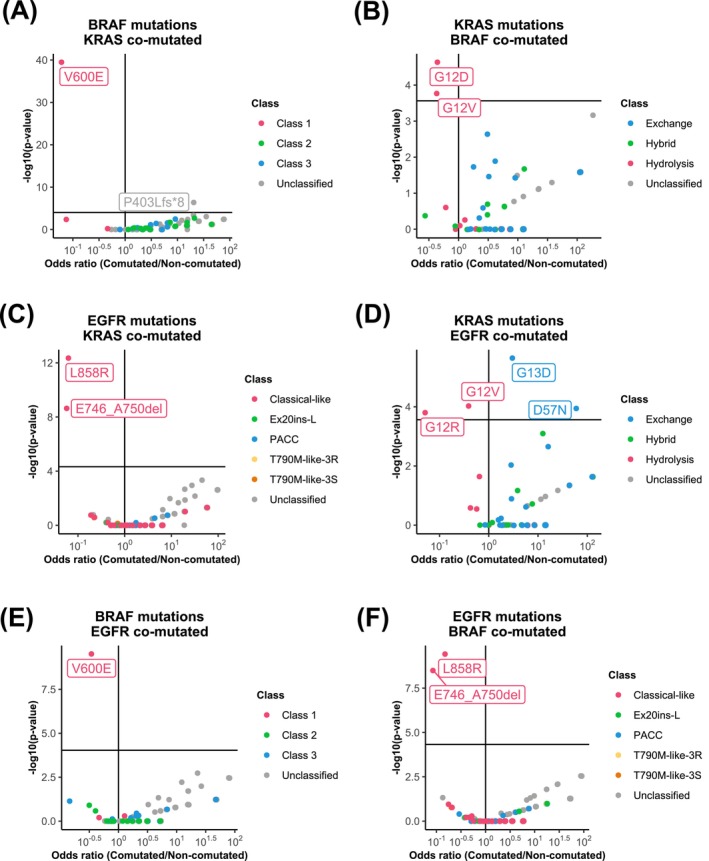
BRAF, KRAS, and EGFR mutations with Mutually Exclusive or Co‐occurring tendencies. Using 2 × 2 contingency tables (as detailed in the methods section), we computed odds ratios (ORs) and two‐sided Fisher's Exact test *p* values. The scatter plots display the ORs of the respective unilaterally class‐assigned variants corresponding to the CO group divided by the ME group against the negative decadic logarithm of the *p* values. The horizontal line borders the statistical significance based on the Bonferroni‐transformed *p* value threshold (as detailed in the methods section), and the vertical line indicates the scenarios with equal frequencies in both CO and ME groups. As such, the upper‐left quarter of each plot represents the mutations that are significantly ME with the comparing genes, and the upper‐right quarter displays variants that are significantly co‐occurring with the comparing genes. Note that compared genes are not class‐assigned, for example, BRAF^V600E^ with KRAS mutations reported in cancer, irrespective of KRAS class. (A) shows the class‐assigned BRAF variants occurring alone or in BRAF∩KRAS. (B) class‐assigned KRAS variants alone or in BRAF∩KRAS. (C) class‐assigned EGFR variants alone or in EGFR∩KRAS. (D) class‐assigned KRAS variants alone or in EGFR∩KRAS. (E) class‐assigned BRAF variants alone or in BRAF∩EGFR. (F) class‐assigned EGFR variants alone or in BRAF∩EGFR. Class assignment of the specific variants is indicated by the color code.

To further investigate the distribution of mutation classes in the context of co‐occurrence, we used Fisher's exact tests to compare the proportions of each class between co‐mutant and single‐mutant groups, showing under‐ or over‐represented classes in the CO groups (Table 1). We then extended the analysis to the level of individual variants, annotated by their respective class (Figure 2).

**TABLE 1:** Co‐occurrence or mutual exclusivity among unilaterally class‐assigned BRAF, KRAS, and EGFR gene variants found in human cancer samples.

Co group	Class	(N) class in co	(N) non‐Class in co	(N) class in non‐co	(N) non‐class in non‐co	Two‐sided Fisher's, *p*	Significant two‐sided	Odds ratio	SE
BRAF∩KRAS	BRAF class								
	I	12	156	1558	983	5.89E‐47	True	0.05	0.30
	II	28	140	191	2350	1.80E‐04	True	2.49	0.22
	III	28	140	223	2318	1.45E‐03	True	2.10	0.22
	KRAS class								
	Hydrolysis	82	86	4811	1463	1.35E‐14	True	0.29	0.16
	Exchange	54	114	946	5328	5.46E‐08	True	2.68	0.17
	Hybrid	13	155	422	5852	5.36E‐01	False	1.20	0.29
EGFR∩KRAS	EGFR class								
	Classical_like	8	140	1363	1486	5.81E‐29	True	0.07	0.36
	Ex20ins_L	0	148	50	2799	1.76E‐01	False	0.19	1.42
	PACC	3	145	164	2685	6.30E‐02	False	0.39	0.55
	T790M_like_3R	0	148	3	2846	1.00E+00	False	2.74	1.51
	T790M_like_3S	1	147	111	2738	4.24E‐02	False	0.25	0.83
	KRAS class								
	Hydrolysis	66	82	4824	1466	1.79E‐16	True	0.25	0.17
	Exchange	54	94	946	5344	2.36E‐10	True	3.26	0.17
	Hybrid	15	133	420	5870	9.81E‐02	False	1.62	0.27
BRAF∩EGFR	BRAF class								
	I	55	106	1514	1033	5.53E‐10	True	0.36	0.17
	II	3	158	215	2332	1.39E‐03	True	0.24	0.55
	III	11	150	240	2307	3.27E‐01	False	0.73	0.31
	EGFR class								
	Classical_like	9	152	1362	1474	3.92E‐31	True	0.07	0.34
	Ex20ins_L	2	159	48	2788	1.00E+00	False	0.90	0.65
	PACC	5	156	162	2674	2.14E‐01	False	0.58	0.44
	T790M_like_3R	1	160	2	2834	1.53E‐01	False	10.60	1.04
	T790M_like_3S	0	161	112	2724	4.09E‐03	False	0.07	1.42

*Note:* Two‐sided Fisher's Exact Test and the 2 × 2 contingency tables, as described in the methods, were used to assess the co‐occurrence or mutual exclusivity of class‐assigned gene variants versus the comparison of gene mutations compiled as a single entity. The analyses were performed on publicly available data extracted via cBioportal, as in Figure 1. **Co Group** represents co‐occurring gene pairs, e.g., BRAF class I, co‐occurring with KRAS mutations reported in human cancer, regardless of KRAS mutation classification. **Classes** refer to classifications described in Supplementary Table S3. Each Co Group is broken down into two class sections, each corresponding to one of the gene components in the specified gene pair. **(N) Class in co** communicates the number of samples that belong to the specified class in the specified co‐occurring gene pair group. **(N) non‐Class in co** represents the number of samples excluding the ones belonging to the specified class in the specified co‐occurring gene pair group. **(N) class in non‐co** displays the number of samples belonging to the specified class when mutually exclusive with the specified component in the gene pair. **(N) non‐class in non‐co** communicates the number of samples that do not belong to the specified class and are found in the mutually exclusive subset concerning the specified gene pair. Based on 2 × 2 contingency tables described in the methods, **Two‐sided Fisher's** *p* values were calculated and displayed. After the Bonferroni adjustment of the significance threshold, whether the **Two‐Sided** test result is **Significant** was determined. The **Odds Ratio** and the standard error (**SE**) are also displayed as inferred.

In BRAF–KRAS co‐mutant samples, class I BRAF mutations were significantly underrepresented, with the canonical V600E variant showing strong mutual exclusivity with KRAS mutations (Table 1, Figure 2A). In contrast, class II and III BRAF mutations appeared more frequently in co‐mutant cases, indicating that atypical BRAF variants are more likely to co‐occur with KRAS mutations. The BRAF^P403Lfs*8^ showed statistically significant co‐occurrence with KRAS mutations (Figure 2A).

We observed mutual exclusivity of hydrolysis‐type KRAS mutations, including G12D and G12V, with BRAF mutations (Table 1, Figure 2B), whereas exchange‐type KRAS mutations were enriched in the co‐mutant group. These results also indicate that mutual exclusivity between BRAF and KRAS is not uniform across mutation subtypes.

In the context of EGFR–KRAS co‐mutations, we observed that classical‐like EGFR mutations, particularly EGFR^L858R^ and EGFR^E746_A750del^, were mutually exclusive with KRAS mutations (Table 1, Figure 2C). The KRAS hydrolysis‐type mutation class—including KRAS^G12R^ and KRAS^G12V^—showed significant mutual exclusivity with EGFR (Table 1, Figure 2D). In contrast, exchange‐type KRAS mutants, such as KRAS^G13D^ and KRAS^D57N^, were enriched in the co‐mutant group. These patterns show that mutual exclusivity and co‐occurrence between EGFR and KRAS are shaped not only by gene identity but also by the functional class of the mutations.

In the case of BRAF–EGFR co‐mutations, we found that both class I and class II BRAF mutations were mutually exclusive with EGFR mutations, whereas class III BRAF mutations did not display mutual exclusivity (Table 1). Classical‐like EGFR mutations were also mutually exclusive with BRAF. The T790M‐like class of EGFR mutations showed a non‐significant trend toward mutual exclusivity with BRAF mutations. At the individual variant level, BRAF^V600E^ is significantly ME with EGFR mutations, whereas EGFR^L858R^ and EGFR^E746_A750del^ variants are significantly ME with BRAF mutations (Figure 2E,F).

Overall, co‐occurrence was rare among class I BRAF, hydrolysis KRAS, and classical‐like EGFR mutations.

### 3.2 Analysis of Cancer Cell Lines Confirms Mutual Exclusivity Patterns Observed in the cBioPortal Tumor Dataset

We next assessed whether the mutual exclusivity and co‐occurrence patterns observed in the cBioPortal dataset were recapitulated in cancer cell lines. We queried 1570 cell lines from the Cancer Cell Line Encyclopedia (Broad 2019). We selected those harboring mutations in BRAF, KRAS, or EGFR. Co‐occurring gene variants are listed in Table S4.

We applied the same analytical approach as in the patient cohorts to evaluate the distribution of mutational classes (Table S5). In contrast to the cBioPortal tumor samples, we did not observe an increased frequency of co‐occurrence of KRAS Exchange or BRAF class III variants in any of the given scenarios. Due to the relatively limited number of available cell lines with CO scenarios, robust statistical conclusions could not be drawn. We only observed a significant mutual exclusivity between class I BRAF and KRAS mutations.

Although the number of co‐mutant cell lines was limited, odds ratio (OR) analysis allowed us to assess directional consistency across datasets. Notably, 17 out of 22 co‐occurrence scenarios showed the same trend in both the cell line and patient (cBioPortal) cohorts (Table S5), supporting the reproducibility of key mutual exclusivity and co‐occurrence patterns.

Notably, none of the CO cell lines harbored concomitant mutations in BRAF class I, KRAS hydrolysis‐type, or EGFR classical‐like variants—the key mutation classes that most strongly associated with mutual exclusivity signals in the cBioPortal dataset. This absence further reinforces the robustness of the observed mutually exclusive patterns across datasets.

### 3.3 Unraveling the Co‐Occurrence Landscape of BRAF, KRAS, and EGFR Mutations in Human Cancer

**FIGURE 3:**
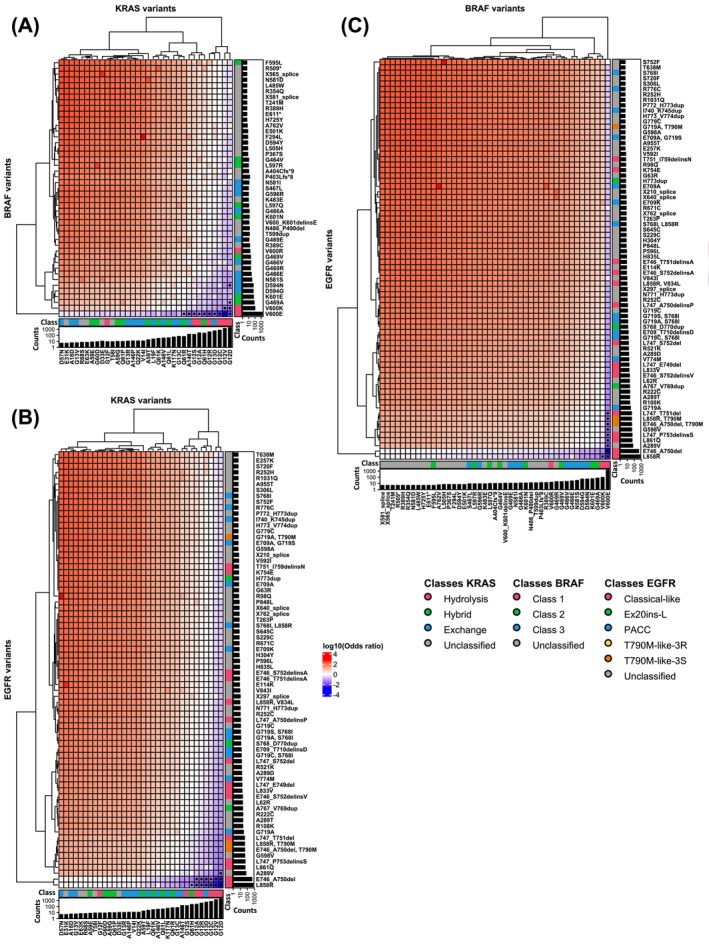
The co‐occurrence landscape of BRAF, KRAS, and EGFR mutations in human cancer. Heatmaps representing co‐occurrence of reported variants of (A) KRAS and BRAF, (B) KRAS and EGFR, or (C) BRAF and EGFR in human cancer observed in at least five patient samples. Log10 odds ratios range from −4 (deep blue, most mutually exclusive) to 4 (deep red, highest co‐occurrence), with white squares indicating equal frequencies in ME and CO. Colored bands on axes indicate gene classes, and black bars represent absolute variant frequencies. Dots denote statistical significance computed by statistical testing with Bonferroni correction and an untransformed *p* value cutoff of 0.05 (as detailed in the methods section). The related tables can be found in Table S9–11.

We next investigated how individual BRAF, KRAS, and EGFR mutations, rather than mutation classes, contribute to co‐occurrence and mutual exclusivity patterns. We were interested in addressing the inter‐class and inter‐type co‐occurrence and mutual exclusivity patterns. To examine these relationships in more detail, we constructed pairwise co‐occurrence heatmaps for each gene pair (BRAF∩KRAS, EGFR∩KRAS, and BRAF∩EGFR), based on the absolute frequency of each variant. For statistical robustness, we included only those variants observed at least five times in human cancer (Figure 3).

In *BRAF*∩*KRAS* (Figure 3A), as in the other gene pairs (Figure 3B,C), mutual exclusivity was not confined to a specific mutation class (Figure 3A). Notably, the most frequent mutations in our cohorts, such as BRAF^V600E^ and KRAS^G12D^, exhibited strong mutual exclusivity, as indicated by low odds ratios. These statistically significant interactions spanned across all BRAF and KRAS classes, suggesting that functional incompatibility extends beyond mutational classes (Figure 3A). Conversely, variant rarity was linked to co‐occurrence, as evidenced by a relatively higher OR among the less frequent mutations. Yet, none of the CO scenarios were statistically significant.

In *EGFR*∩*KRAS*, EGFR^E746_A750del^ and EGFR^L858R^ mutations showed pronounced mutual exclusivity with the common KRAS variants (Figure 3B). All KRAS classes were represented among mutually exclusive pairs. Among the classified EGFR variants, only Classical‐like was represented in highly ME scenarios. Among EGFR mutations, significant ME interactions were restricted to Classical‐like variants, with the exception of EGFR^A289V^, an atypical variant predominantly found in glioblastoma [40] but also reported in other cancer types, such as colorectal cancer [41], which also showed strong and statistically significant exclusivity with KRAS^G12D^.

In *BRAF*∩*EGFR*, we identified specific instances of Classical‐like, T790M‐like‐3S, or unclassified EGFR mutations, EGFR^G598V^ and EGFR^A289V^, to be significantly mutually exclusive with BRAF^V600E^ (Figure 3C).

Overall, our co‐occurrence matrices revealed some novel ME scenarios. From a broader perspective, we unraveled the pan‐cancer landscape of mutual exclusivity among BRAF, KRAS, and EGFR mutations. This landscape showcases the link between mutational frequency and mutual exclusivity.

To further investigate the biological relevance of these mutually exclusive variant pairs, we analyzed samples with a significant ME. We identified only nine such samples. Allele frequency (AF) data were available for six of these nine cases. In four of these, one mutation of the pair, typically KRAS, had an AF below 10%, which could indicate subclonality, spatial heterogeneity within the tumor, or sample contamination (Table S6). Given the limited number of cases and partial availability of AF data, sample‐level characteristics offered insufficient resolution to further clarify the nature of these co‐mutant events. Notably, the only BRAF∩EGFR co‐mutant sample was from a patient receiving EGFR‐targeted therapy, consistent with the reports that BRAF mutations can emerge as a resistance mechanism in patients receiving EGFR‐targeted therapy [24, 25, 42].

### 3.4 Mutual Exclusivity and Co‐Occurrence in a Belgian Cancer Patients' Dataset

To validate our findings from the cBioPortal dataset, we analyzed a manually curated cohort of 2714 tumor samples from cancer patients who underwent targeted next‐generation sequencing. While cBioPortal is extensive, it lacks standardized information on tumor type and variant‐level allele frequency, and it provides variants indiscriminately regardless of their biological or clinical relevance. In contrast, the Belgian dataset includes expert‐reviewed variant interpretation and classification, applying a national guideline, resulting in the classification of all variants as (likely) pathogenic, (likely) benign, or of unknown significance. This enabled us to assess co‐occurrence and mutual exclusivity patterns specifically among variants with a defined or suspected biological impact.

**FIGURE 4:**
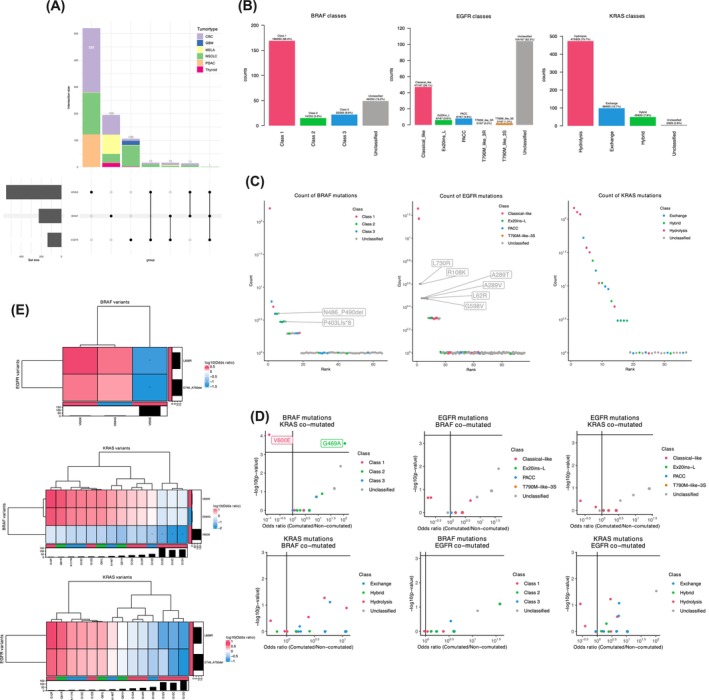
Mutual Exclusivity and Co‐occurrence in a Belgian cancer patients' dataset. The Belgian cancer NGS dataset encompasses curated variants of 2714 unique samples from patients with solid tumor types across several tissue types, collected between July 1, 2019, and April 24, 2023. The dataset includes 260 BRAF, 636 KRAS, and 184 EGFR variants (SNVs, splice site variants, and short indels), of which, respectively, 63, 37, and 115 are unique gene variants. In (A), an Upset plot displays the distribution and overlap of 231 BRAF, 568 KRAS, and 154 EGFR‐variants in 1711 patient samples among six tumor types, each harboring at least 5% of all BRAF, KRAS, or EGFR variants identified in the complete Belgian dataset (NSCLC; Non‐small Cell Lung Cancer, CRC; Colorectal cancer, PDAC; Pancreatic Ductal Adenocarcinoma, MELA; Melanoma, GBM; Glioblastoma, and Thyroid cancer). Variants are listed in Table S12. The frequencies of gene‐specific variants across the six tumor types align with previously reported findings. The CRCs, NSCLCs, and PDACs are recurrently found to harbor KRAS variants [43]. In NSCLC, this is followed by EGFR variants and subsequently BRAF variants. In CRC, however, this order is reversed [18, 19]. BRAF variants are typically associated with thyroid cancer and melanoma [18]. In glioblastomas, EGFR is often affected by variants in the extracellular region of the protein [44]. The occurrence of variants in specific gene pairs is mainly restricted to NSCLC and CRC; BRAF∩KRAS in six NSCLCs and eight CRCs, BRAF∩EGFR in four NSCLCs, nine CRCs, and three melanomas, KRAS∩EGFR in nine NSCLCs, six CRCs, and one endometrial carcinoma (not shown in Figure 4a). One NSCLC tumor was found with coexistence of mutations in all three mentioned genes. In (B), bar charts show the relative frequencies of BRAF, KRAS, and EGFR mutational classes as percentages across all 2714 Belgian cancer samples tested. These frequencies closely align with those identified in the cBioPortal patient dataset, apart from the EGFR class distribution. For EGFR, we found fewer Classical‐like variants (28.1% vs. 45.4%) but a higher frequency of unclassified variants (62.3% vs. 43.6%). One possible explanation for this variation could be attributed to the assumed composition of the Belgian test population, which is expected to include a smaller proportion of patients with Asian ethnicity. However, it should be noted that no demographic data were included in the analysis. The Belgian dataset also encompassed glioblastoma cases, which often feature EGFR mutations within the extracellular domain, probably contributing to the slightly increased occurrence of unclassified EGFR variants (3% Belgian GBM vs. 1% cBioPortal GBM) [44]. In (C–E), we provide graphs, scatter plots and heatmaps (for mutations that occur at least 5 times in the dataset) showcasing the entire Belgian dataset, all generated analogously as for Figures 1b, 2, and 3, respectively. In (**C**), several unclassified variants are among the top 10 variants. For BRAF these include BRAF^N486_P490del^ and BRAF^P403Lfs*8^, and for EGFR they include EGFR^A289V^, EGFR^A289T^, EGFR^G598V^, EGFR^R108K^, EGFR^L62R^ (all recurrent in GBM), and also EGFR^L730R^ (primarily detected in NSCLC). In (D), we exclusively observe significant scenarios involving KRAS mutations, such as with Class I BRAF^V600E^ in the ME pattern and with BRAF^G469A^ in the CO pattern. In (E), the co‐occurrence heatmaps indicate CO scenarios involving less frequent mutations (without statistical significance), while ME scenarios exhibit significance for specific mutation types.

First, we examined the distribution of BRAF, KRAS, and EGFR mutations across tumor types and mutation frequencies to contextualize potential ME and CO scenarios. To explore how mutation patterns relate to tumor type and variant frequency, we visualized the distribution of BRAF, KRAS, and EGFR mutations across six major tumor types in the Belgian cohort (Figure 4A). The observed frequencies are consistent with those reported in the literature, supporting the dataset's representativeness. Notably, co‐occurring mutations within gene pairs were largely confined to specific tumor types, underscoring the influence of tissue context on co‐mutation patterns.

We assessed the frequency of BRAF, KRAS, and EGFR mutation classes across all tumor types (Figure 4B). The overall class distributions were similar to those observed in the cBioPortal dataset. We found two atypical variants within the top ten BRAF variants, namely BRAF^N486_P490del^ and BRAF^P403Lfs*8^ (Figure 4C). The BRAF^P403Lfs*8^ variant significantly co‐occurred with KRAS mutations in the cBioPortal dataset and was found to co‐occur with KRAS in three Belgian cases. Notably, co‐occurrence cases were in mismatch repair‐deficient cancers. Several other atypical variants were also among the top ten most frequent EGFR variants. These include EGFR^A289V^, EGFR^A289T^, EGFR^G598V^, EGFR^R108K^, and EGFR^L62R^, which are recurrently found in glioblastoma.

We next examined the relationship between class‐assigned variants of one gene and all variants of the other genes (Figure 4D). Among the EGFR classes, no significant patterns of co‐occurrence or mutual exclusivity with KRAS or BRAF variants were observed. Similarly, KRAS and BRAF classes did not show significant associations with EGFR variants. As in the cBioPortal analysis, class I BRAF^V600E^ remained significantly mutually exclusive with KRAS mutations. Class II BRAF^G469A^ showed a statistically significant co‐occurrence with KRAS mutations; however, this was based on only three samples in the Belgian cohort, limiting interpretability. These BRAF^G469A^ co‐occurrences involved KRAS^G12V^ and KRAS^G12C^ in colorectal cancer and KRAS^G12A^ in non‐small cell lung cancer. Notably, none of the co‐occurring pairs included KRAS^G12D^, which was consistent with our findings in the cBioPortal dataset (Figure 3A).

Finally, we generated co‐occurrence heatmaps for each of the three CO scenarios in the Belgian dataset (Figure 4E). As observed in the cBioPortal analysis, co‐occurrence primarily involved less frequent variants and did not reach statistical significance. Mutual exclusivity patterns were also not confined to specific mutation classes. In the EGFR∩KRAS and EGFR∩BRAF comparisons, the Classical‐like variants EGFR^E746_A750del^ and EGFR^L858R^ showed a strong trend toward mutual exclusivity with Hydrolysis‐class KRAS^G12V/D/C^ variants, and EGFR^L858R^ was significantly mutually exclusive with class I BRAF^V600E^. In the KRAS∩BRAF group, class I BRAF^V600E^ was significantly mutually exclusive with both Hydrolysis‐class KRAS^G12V/D/C^ and the Exchange‐class KRAS^G13D^ variant.

Overall, in line with our prior analysis, gene classifications showed significant variability in CO patterns relative to other gene variants. In this regard, discrepancies were observed between our findings in the cBioPortal analysis and those in the Belgian cancer dataset. Once again, we observed that a variant‐specific landscape might offer more informative insights into co‐occurrence and mutual exclusivity patterns than gene classifications.

### 3.5 Association Between Mutation Co‐Occurrence Patterns and Overall Survival in Colorectal and Lung Cancer

To assess whether co‐occurrence and mutual exclusivity patterns among BRAF, KRAS, and EGFR mutations are associated with clinical outcome, we analyzed overall survival data from 6250 colorectal cancer (CRC) and 9418 non‐small cell lung cancer (NSCLC) patients available via cBioPortal. Only cases harboring at least one mutation in BRAF, KRAS, or EGFR were included. We stratified patients into mutually exclusive and co‐occurring mutation groups (at the gene level) and generated Kaplan–Meier survival curves to compare five‐year overall survival across these categories (Figure S2, Table S7A–F).

Across most comparisons, no statistically significant difference in survival was observed. However, NSCLC patients with EGFR‐KRAS co‐mutations showed a statistically significant reduction in overall survival compared to those with EGFR mutations alone (see Figure S2 legends).

### 3.6 Exogenous Induction of Oncogenes Compromises Cellular Fitness in Cells Harboring Mutually Exclusive Endogenous Partners

To distinguish between stochastic absence and negative selection as underlying causes of mutual exclusivity, we tested whether enforced expression of a second, mutually exclusive oncogene impairs cellular fitness. This approach aimed to evaluate whether the lack of co‐occurrence in tumors reflects a selective disadvantage for cells harboring a mutually exclusive mutation pair.

To investigate this, we transduced intestinal (LS513) and lung adenocarcinoma (PC‐9) cell lines with doxycycline‐inducible lentiviral vectors encoding either wild‐type or oncogenic variants of BRAF or EGFR cDNA, linked to green fluorescent protein (GFP). A GFP‐only construct served as a control, as in a previous study [11] (Table S13). Although wild‐type genes themselves have been shown to cause functionally antagonistic effects when co‐expressed with a second oncogene [32], we decided to include them as extra controls alongside mutant constructs.

We suspected that, over time, cells with low or silenced transgene expression might gain a growth advantage within the polyclonal pool, due to the leaky nature of the doxycycline‐inducible systems [45] and potential growth‐suppressing effects of the second oncogene, even in the absence of doxycycline treatment. To minimize this bias, we prioritized analyzing early passages after infection for the most reliable results. After stable genomic integration and validation of the inducible transgene expression in both cell lines (Figure S3A–D), we assessed cell confluency in response to doxycycline‐induced expression of the second oncogenic mutation using live‐cell imaging.

Notably, doxycycline is known to exert growth‐inhibitory effects on various cell types in vitro [46, 47] (independent of its role in inducing transgene expression in our case). Consistent with this, live‐cell imaging revealed growth inhibition in GFP‐expressing control cell lines upon late exposure to doxycycline.

**FIGURE 5:**
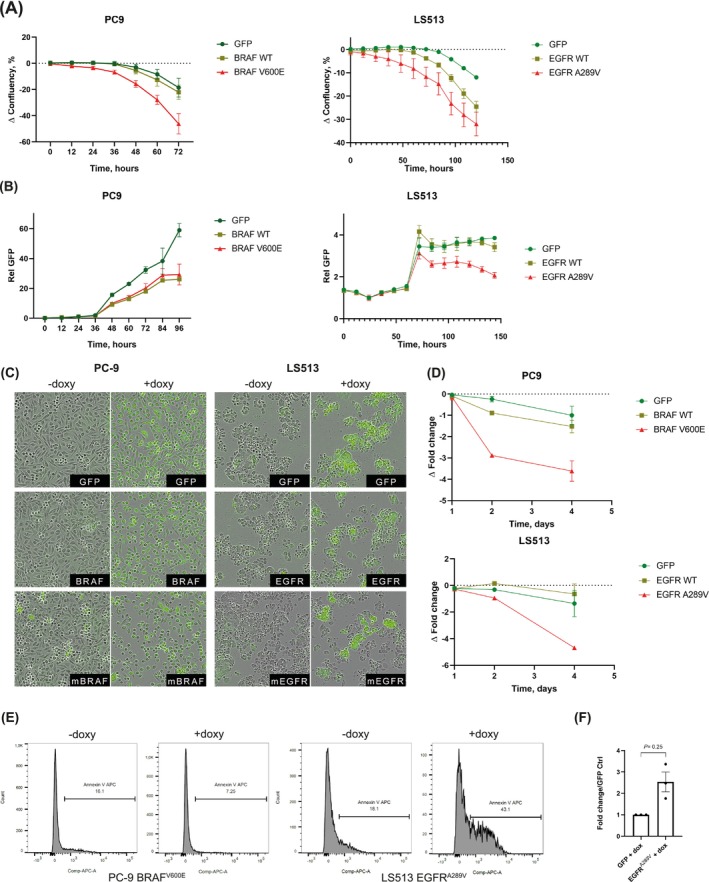
Exogenous induction of oncogenes compromises cellular fitness in cells harboring mutually exclusive endogenous partners. (A) Analysis of cell confluency using live video imaging. Delta confluency was calculated for each time‐point as the difference between the confluency of doxycycline‐induced and control non‐treated cells. The mean values and standard deviations from three technical replicates are shown. (B) Relative intensity of GFP measured in the GFP‐positive cell population using live video imaging. The values shown were calculated for doxycycline‐treated samples as the ratio of the values corresponding to GFP‐positive cells at each time‐point to the values acquired for GFP‐positive cells 24 h after treatment start, when GFP signal was clearly detectable, but the effect of the transgene's expression was expected to be minimal. (C) Representative images of PC‐9 (day 4) and LS513 cells (day 6) obtained from the IncuCyte live cell imaging. (D) Cell viability assay. Cells were seeded in 96‐well plates with the same density as in (A), and viability was measured at the indicated time‐points using the CellTiter‐Glo, according to the manufacturer's protocol. Background luminescence was subtracted; the values were normalized to day 1 control (non‐induced) samples to calculate the signal fold change. Next, the difference in fold change between doxycycline‐treated and non‐treated cells was calculated (D Fold change). The mean values and standard deviations from 3 technical replicates are shown. (E) Apoptosis was detected in the indicated cell lines using the Annexin V kit (MabTag) according to the manufacturer's protocol and measured by flow cytometry at day 7 after induction. (F) Quantification of the ratio between double‐positive GFP‐Annexin V populations in LS513 control (GFP) and EGFR^A289V^ cell lines after 7 days of doxycycline treatment (three independent experiments, Wilcoxon Signed Rank Test's *p* value is displayed).

In PC‐9 cells, which endogenously harbor the EGFR^E746_A750del^ mutation, doxycycline treatment in the BRAF^V600E^ inducible cell derivative substantially reduced proliferation compared to the doxycycline inducible GFP control line. The PC‐9 cells with induced BRAF^V600E^, identified as GFP positive, expanded slowly and plateaued after approximately 3.5 days, showing visibly reduced proliferation (Figure 5A–C, Figure S3A,B, Movies S1 and S2). Although BRAF^V600E^ induction impaired proliferation, we did not observe an increase in Annexin V/APC positivity (Figure 5E,F).

In LS513 cells, which natively carry homozygous KRAS^G12D^, induction of EGFR^A289V^ led to a progressive decline in GFP‐positive cells, starting around day 3, consistent with impaired proliferation. This was accompanied by morphological changes, including cell shrinkage and detachment (Figure 5B,C, Figure S4A–B, Movies S3 and S4). FACS analysis revealed that EGFR^A289V^ induction increased Annexin V/APC positivity, indicating a higher proportion of dying cells (Figure 5D–F).

In both cell lines, viability assays corroborated the live cell imaging observations (Figure 5D).

Our data demonstrate that exogenous induction of oncogenes reduces cellular fitness when mutually exclusive endogenous oncogenic partners are present. These findings support the notion that certain oncogenic mutations are mutually exclusive in cancer because their combined expression imposes a selective disadvantage on tumor cell viability.

### 3.7 Epistasis‐Aware Validation of Variant‐Level Mutual Exclusivity

So far, we have assessed mutual exclusivity across datasets using contingency table‐based methods, such as Fisher's exact test (FET). Systematic literature review (Table S8) and new experiments further unraveled that our discovered variant‐specific landscape of mutual exclusivity among BRAF, EGFR, and KRAS oncogenes overlaps with functionally antagonistic mutant pairs. However, a limitation of contingency table–based analyses, such as FET, is that they do not explicitly model epistasis, defined as non‐independent effects of mutations on cellular fitness, whereby the combined impact of two variants deviates from the expectation based on their individual effects. In cancer, such epistatic interactions frequently arise within oncogenic signaling pathways and can manifest as mutual exclusivity when co‐occurring mutations provide no additional selective advantage or are deleterious. CancerEffectSizeR [37] (CES) explicitly models variant‐level selection and epistatic interactions, thereby providing an orthogonal framework for evaluating mutually exclusive mutation pairs identified by our FET‐based pipeline.

To maximize the strengths of available high‐quality data, while CES was not applied to our full cohort due to computational constraints, the comparison utilized the PCAWG pan‐cancer whole‐genome dataset featuring 2922 well‐characterized tumors, 413 of which carried mutations in KRAS, EGFR, or BRAF (106 KRAS‐mutant, 273 EGFR‐mutant, and 49 BRAF‐mutant samples). Applying both methods to this subset yielded highly concordant *p* values (Figure S5A; Table S14), with the only statistically significant variant pair—KRAS^G12D^ and BRAF^V600E^—corresponding to the strongest mutually exclusive association identified by our FET analysis in the full dataset (Figure S5B; Table S15). This reflects both methodological agreement and the limited statistical power of the smaller cohort.

We next examined how the 38 variant pairs identified as significantly mutually exclusive in the full cohort behaved under CES in the PCAWG dataset (Figure S5B; Table S15). Eighteen pairs could not be evaluated because they involved EGFR^L858R^, which is absent from the PCAWG cohort, or the EGFR^E746–A750 deletion^, which cannot be robustly handled by CES as it aggregates multiple distinct deletion events into a single variant. Among the remaining pairs, interestingly, the most significant associations from the full analysis also showed the lowest *p* values in the CES analysis, whereas reduced concordance for other pairs was accompanied by marked differences in variant frequencies between datasets, indicating limited representation of rarer variants in the PCAWG cohort.

## 4 Discussion

Overall, Type I BRAF, Hydrolysis KRAS, and Classical‐like EGFR class mutations, all known as strong activators of the ERK pathway, are less likely to co‐occur with each other [18, 19, 20]. Our co‐occurrence heatmaps with bilateral class/variant assignments revealed several novel ME scenarios. For instance, the first part of our analyses concluded that BRAF class II is enriched in the BRAF∩KRAS group, in line with a recent report by Zhao et al. [21]. However, heatmaps revealed mutual exclusivity between the well‐studied class II BRAF^G469A^ and KRAS^G12D^. At the level of individual variants, only BRAF^P403Lfs*8^ showed statistically significant co‐occurrence with KRAS mutations. This variant is poorly characterized and is widely presumed to be a passenger mutation, commonly found in tumors with high mutational burden associated with mismatch repair deficiency [48]. The variant has also shown a growth‐inhibitory effect when overexpressed in Ba/F3 and MCF10A cells [49].

Additionally, we identified previously unknown ME pairs involving atypical EGFR mutations and BRAF and KRAS mutations, which warrant further preclinical investigation. The ME scenarios showed statistical significance, whereas co‐occurring pairs did not. In a pan‐cancer analysis of mutual exclusivity among somatic mutations, Canisius et al. propose that mutual exclusivity in cancer is a product of active biological phenomena, while co‐occurrences are somewhat random [3]. As such, our findings could align with those of Canisius et al. [3]. Conversely, the lack of statistical significance may be due to the infrequency of such scenarios. Furthermore, it is crucial to consider an inherent limitation of pan‐cancer analyses, including our study and that of Canisius et al. in which the significance of context‐dependent associations might be overlooked because all tissue types are conflated within a single analytical framework.

We reviewed precedent evidence to determine whether redundancy in function alone underlies the mutual exclusivity of some mutations, or whether an active biological mechanism limits or even excludes such co‐occurring events. To tailor our revisit of the precedent literature to the ME scenarios in our current findings, we systematically reviewed the literature for studies that have modeled concomitant induction of the very oncogenic variants as identified in this study, in the same cell (Table S8). Briefly, in different preclinical lung cancer models, co‐expression of BRAF^V600E^∩KRAS^G12D^ [14], KRAS^G12V^∩EGFR^L858R^ [10], EGFR^exon19del^∩KRAS^G12C^ [10], and EGFR^L858R^∩KRAS^G12V^ [22] has been investigated. Across all these studies, the co‐induction of two oncogenes could not be tolerated in the same cell. The coexistence of both oncogenes was synthetically lethal or led to senescence [10, 14, 22]. In these cases, the observed phenomenon was at least partly attributed to supra‐physiological levels of MAPK signaling [10, 14, 22].

Although rare, the coexistence of EGFR and BRAF mutations has been reported in NSCLC [50]. The emergence of secondary BRAF mutations in NSCLC patients treated with EGFR inhibitors has been documented [24, 25]. In at least one case, a treatment‐naïve primary tumor harbored both EGFR exon 19 deletion and BRAF^V600E^ mutations [51]. Notably, the mutation abundances for both genes were low, 10.33% for EGFR and 3.51% for BRAF [51]. These findings suggest that such co‐occurrences may not exist in the same cell. Another study [52], which retrospectively screened 423 NSCLC tumors, reported a case with EGFR^L858R^ and BRAF^V600E^.

On the other hand, we did not find direct biological evidence linking the mutual exclusivity of oncogenic BRAF and EGFR mutations to senescence or synthetic lethality. Two experimental studies have been inspired by the emergence of BRAF mutation in EGFR‐mutant and relapsed lung cancers as they become resistant to EGFR‐targeted therapies [24, 25]. We have previously postulated that BRAF^V600E^ and classical EGFR mutants would not be tolerated in the same cell [32]. In this study, we demonstrate that BRAF^V600E^ expression in cells with endogenous EGFR^exon19del^ leads to growth inhibition. Moreover, we provide unprecedented evidence for atypical EGFR^A289V^, demonstrating that its induction in cells with endogenous KRAS^G12D^, its ME variant partner, leads to growth inhibition.

Therefore, as previous literature suggests, active biological phenomena such as senescence and synthetic lethality can limit the likelihood of a few CO events described in this study, at least in some BRAF∩KRAS and EGFR∩KRAS pairs. We present similar but unprecedented experimental evidence about EGFR∩BRAF and EGFR∩KRAS scenarios.

Perhaps we could consider that the redundancy of functions and synthetic lethality are not necessarily conflicting scenarios, as the latter can involve excessive activity of two redundant functions. As such, they might both contribute to the rise of mutual exclusivity. Yet further benchwork and collective effort are essential to elucidate the mechanisms underlying the novel ME pairs reported in this study.

Based on the available patient dataset, we could not determine whether BRAF, KRAS, and EGFR mutations had occurred in the same cells in the CO groups. Yet, our analysis of cell lines could address this question. In a study by Gularte‐Mérida et al. [53], leveraging single‐cell RNA sequencing of tumor samples, the authors report that, albeit at very low frequency, BRAF^V600E^ and KRAS^G12D^ events can coexist in the same cell in a cohort of treatment‐naive colorectal cancer patients. Notably, this co‐occurrence was accompanied by a compromise of allelic imbalance, a phenomenon that favors increased oncogene dosage through different mechanisms in cells harboring a single oncogene (RAS or BRAF) [54]. Once again, these findings align with the concept of *oncogene overdose* [55]. In general, human cells, including cancer cells, cannot tolerate exceeding a certain threshold of ERK pathway activity [56, 57, 58].

In line with Gularte‐Mérida et al.'s study [53], we did not find a significant difference between CO and ME groups in CRC patients. However, in NSCLC, EGFR∩KRAS patients had poorer OS than ME mutant EGFR patients. We found that EGFR mutations within the EGFR∩KRAS co‐occurrence are enriched with atypical variants. These variants are characterized by low or absent sensitivity to standard EGFR‐targeted TKIs [19]. Conversely, EGFR variants in the ME groups have a high frequency of classical‐like variants. These variants, by contrast, are generally responsive to EGFR TKIs [19]. Consequently, the difference in variant frequency between the ME and CO groups, as well as the relatively good prognosis of NSCLC patients with typical EGFR mutations [59, 60], may explain the diminished OS among EGFR∩KRAS NSCLC patients. As previously reported, EGFR TKIs such as gefitinib yield worse outcomes in patients with wild‐type EGFR than chemotherapy [61]. One could postulate that KRAS can drive downstream signaling pathways in such a way that reduces EGFR dependence, leading to loss of sensitivity to EGFR TKIs. Moreover, oncogenic KRAS mutations have been linked to a more aggressive phenotype and therapy resistance, which may independently contribute to poorer overall survival [62]. It remains unclear whether patients underwent resequencing after TKI resistance. Additionally, a higher incidence of genetic instability among CO patients may lead to the accumulation of multiple mutations in the CO group and even to the development of resistance, regardless of the therapy employed.

We emphasize that, in our analysis, we could not account for disease stage, a significant determinant of OS. Nevertheless, it is essential to note that our OS analyses did not acknowledge other potential confounding factors, including patient characteristics, treatment type, and variant‐specific differences. A comprehensive multivariate analysis, ideally involving larger cohorts of CO patients, is warranted to draw a definitive conclusion about the inherent impact of the co‐occurrence of these oncogenes on clinical outcomes.

Our CO matrices in the Belgian dataset confirmed the ME scenarios, provided that both variants had previously been included in the analysis of the available sample data in cBioPortal. As we further observed in the Belgian cancer patient dataset, all three instances of BRAF^G469A^ occurred in patients with KRAS‐activating variants. This co‐occurrence and further co‐expression, if it happens in the same cell, contradict prior conclusions that have classified BRAF^G469A^ as RAS‐independent [18]. It is worth mentioning that none of the co‐occurring KRAS variants were KRAS^G12D^. This underscores the robustness of our approach in revealing variant‐specific mutual exclusivity in human cancer.

Furthermore, we highlight several EGFR variants that had previously been overlooked for their significance. These variants were recurring in both publicly available and our clinical datasets.

Beyond the mutation types, we did not address the roles of other players that may affect mutual inclusivity or exclusivity among BRAF, EGFR, and KRAS mutations. Such players could include ERK effectors and regulators. Regarding certain variants, such as EGFREx20ins‐L and PACC class members, our consulting dataset was limited, disabling us to draw robust statistical conclusions. Moreover, we did not address the chronological order of gene mutations in patients with CO gene pairs.

Further, we compared our pan‐cancer findings with previous reports on specific cancer types. Such studies offer greater context specificity than ours. In some, no CO mutations were observed. This is likely due to limited sample sizes or enrichment for context‐specific factors (e.g., cancer type or patient ethnicity). KRAS and BRAF mutations were generally mutually exclusive in colorectal cancer, colorectal adenoma, and small intestinal adenocarcinoma samples [63, 64, 65, 66], and a single thyroid carcinoma sample set [67]. One study of metastatic colorectal cancers [68], showed a low frequency of EGFR and BRAF mutations compared to KRAS mutations. In two colorectal cancer cases, BRAF^G466R^ was reported to co‐occur with KRAS^G12V^ or KRAS^G12S^ [66]. Note that, in our analysis, the number of BRAF^G466R^ cases was fewer than five, the threshold we set in our study. In line with our findings, in NSCLC, KRAS co‐mutations were reported to be ME with BRAF class I but not with class II or class III [69].

KRAS and EGFR co‐mutations have been reported. For instance, the co‐existence of KRAS^G12C^ and EGFR^exon19del^ is reported in lung cancer [70]. Whether these mutations coexist in the same cell remains uncertain.

In one study with access to VAF data, the co‐mutations likely represented distinct subclonal populations, not the same tumor cells [71]. Nevertheless, single‐cell analysis is needed to confirm this interpretation. In such rare cases, we still do not know whether, if expressed, both mutant proteins are functionally active or whether one overshadows the other. Still, one cannot rule out the possibility that, in rare subclones, cells can rewire pathways in unexpected ways to remain dependent on a single oncogene.

A key limitation of our primary approach is the use of empirical measures of mutual exclusivity and co‐occurrence without accounting for gene‐ and trinucleotide‐specific mutation rates or tumor‐specific mutagenic processes, which can confound observed patterns. As such, these findings should be interpreted as preliminary associations, and future work incorporating mutation‐rate‐aware models will be essential to more accurately assess selective interactions. Advanced pipelines [37, 72, 73, 74] have been developed to correct for confounding factors such as mutation rates and genomic context [75, 76, 77, 78, 79, 80, 81, 82, 83, 84, 85, 86, 87]. To address potential limitations of contingency table‐based methods like FET, which do not model epistasis or adjust for mutation rates, we orthogonally validated our strongest FET‐identified mutual exclusivity signals using the epistasis‐aware CancerEffectSizeR (CES) [37] on the PCAWG pan‐cancer cohort [36], confirming high concordance for key variant pairs. Overall, these results demonstrate that the strongest mutually exclusive variant pairs identified by our FET‐based pipeline are consistent with epistasis‐aware modeling. Reduced concordance for less frequent variants is perhaps driven by cohort size and variant coverage rather than methodological discrepancies.

Our findings on classes with diverse co‐occurrence patterns emphasize the need to revise the mutational classifications for BRAF, KRAS, and EGFR. In CO mutation scenarios, context‐ and mutual dependencies should be investigated.

In recent years, we learned about the vulnerability of ERK‐associated cancer cells to ERK pathway agonism [9, 55, 58, 88]. Sensitivity to ERK pathway agonism is perhaps a hallmark of ERK‐associated cancers, and particularly those harboring classical BRAF, EGFR, or KRAS mutations [32]. This emerging field [89] has transformed from mere conceptualization to the preclinical exploration of ERK pathway activators [56, 88, 90, 91, 92] for anti‐cancer targeting (reviewed here [32]). The variant‐specific landscape of mutual exclusivity identified in this study, besides being a reference repository for precision oncology, sheds light on the potential utility [9] of exploiting the shared expression of two mutually exclusive and synthetically lethal genes. This knowledge can facilitate the prediction of synthetically lethal scenarios and inform preclinical investigations for personalized treatments. Accordingly, we have proposed a target discovery framework for potential ERK pathway agonists [32].

The mechanisms elicited by the coincidence of mutually exclusive events described in this study warrant investigation, as they can reveal unexplored vulnerabilities and novel therapeutic opportunities in ERK‐related cancers.

## Author Contributions

**Maxim Noeparast:** conceptualization, data curation, formal analysis, funding acquisition, investigation, methodology, project administration, resources, supervision, validation, visualization, writing – original draft, writing – review and editing. **Freya Vaeyens:** data curation, formal analysis, investigation, methodology, validation, visualization, writing – original draft, writing – review and editing. **Jan‐Patrick Hetzel:** conceptualization, data curation, formal analysis, investigation, methodology, software, validation, visualization, writing – original draft, writing – review and editing. **Khaldoon Abdullah:** formal analysis, investigation, methodology, writing – review and editing. **Carolien Eggermont:** data curation, investigation, writing – original draft, writing – review and editing. **Catharina Olsen:** data curation, formal analysis, investigation, methodology, software, visualization. **Marco Mernberger:** data curation, formal analysis, software, visualization, writing – review and editing. **Ken Maes:** investigation, writing – review and editing. **Jelle Vlaeminck:** investigation, writing – review and editing. **Rainer Claus:** investigation, writing – review and editing. **Dries Vanisterbecq:** investigation, writing – review and editing. **Frederik Hes:** project administration, resources, supervision, writing – review and editing. **Martin Pichler:** data curation, formal analysis, funding acquisition, investigation, resources, writing – review and editing. **Philippe Giron:** conceptualization, data curation, formal analysis, funding acquisition, investigation, methodology, project administration, resources, supervision, writing – original draft, writing – review and editing. **Oleg Timofeev:** conceptualization, data curation, formal analysis, funding acquisition, investigation, methodology, project administration, resources, supervision, writing – original draft, writing – review and editing.

## Funding

The work performed by the Centre for Human Genetics (Belgium) was co‐funded by the Wetenschappelijk Fonds Willy Gepts of the UZ Brussel/VUB and the UZ Brussel Foundation. Oleg Timofeev had received the following: The Federal Ministry of Education and Research (BMBF) grant 161L0279A and German Research Foundation (DFG) grant Ti1028/2‐1. Maxim Noeparast had received the following: Fonds Wetenschappelijk Onderzoek (FWO) – Vlaanderen, Belgium, 12Y0120N, and his research was further financed by the Department of Hematology and Clinical Oncology, University Medical Center, and Medical Faculty Augsburg University, Augsburg, 86156, Germany.

## Ethics Statement

Ethical approval was obtained from the Medical Ethics Committee of UZ Brussel/VUB for the study of NGS data from Belgian diagnostic cancer patients (EC‐2023‐040).

## Conflicts of Interest

The authors declare no conflicts of interest.

## Supporting information


**Movie S1:** Representative movie of PC‐9 cells stably transduced with the BRAFV600E oncogene, without doxycycline (day 0–6).

**Movie S2:** Representative movie of PC‐9 cells stably transduced with the BRAFV600E oncogene, treated with 1 μg/mL doxycycline (day 0–6).

**Movie S3:** Representative movie of LS513 cells stably transduced with the EGFRA289V oncogene, without doxycycline (day 0–6).

**Movie S4:** Representative movie of LS513 cells stably transduced with the EGFRA289V oncogene, treated with 1 μg/mL doxycycline (day 0–6).

**Figure S1:** Frequency of cancer types among the publicly available dataset queried for the current study.
**Figure S2:** Kaplan–Meier curves for assessing Overall Survival rates in Colorectal Cancer and Non‐small Cell Lung Cancer patients' groups with BRAF, KRAS, or EGFR Mutations.
**Figure S3:** ijc70558‐sup‐0005‐Figure S1‐S5.pdf.
**Figure S4:** ijc70558‐sup‐0005‐Figure S1‐S5.pdf.
**Figure S5:** Epistasis‐aware validation of variant‐level mutual exclusivity using CancerEffectSizeR.

**Table S1:** Gene‐specific variants found in human cancer samples and corresponding tissue types extracted from cBioportal and used in this study.
**Table S2:** Sequencing statistics of the Belgian cancer NGS dataset (based on reference genome GRCh37).
**Table S3:** Classification of BRAF, KRAS, and EGFR mutations acknowledged in this study.
**Table S4:** Co‐occurring Gene variants identified in the same cell line.
**Table S5:** Co‐occurrence or mutual exclusivity among unilaterally class‐assigned BRAF, KRAS, and EGFR gene variants found in human cancer cell lines.
**Table S6:** Samples with yet co‐occurrence of ME scenarios with statistical significance and their related tissue types AF.
**Table S7:** (a–f) Overall Survival data belonging to CRC and NSCLC patients with BRAF, KRAS, and EGFR mutations stratified to ME or CO groups and adjusted to 5 years.
**Table S8:** Precedent studies reveal that synthetic lethality and senescence lie behind mutual exclusivity among some oncogenic BRAF, KRAS, and EGFR events.
**Table S9–11:** Tables related to Figure 3.
**Table S12:** Gene‐specific variants identified in the Belgian dataset of six tumor types (4).
**Table S13:** Cell lines used in vitro experiments.
**Table S14–15:** Table related to Figure S5.

## Data Availability

Data sources and handling of the publicly available datasets used in this study are described in the Materials and Methods. The R script developed during this study can be found at https://github.com/HetzBioRepo/Variant‐Specific‐Landscape‐of‐Mutual‐Exclusivity. Further details and other data that support the findings of this study are available from the corresponding authors upon request.

## Endnotes

^1^
RTK/RAS/RAF (receptor tyrosine kinase/rat sarcoma/rapidly accelerated fibrosarcoma), also known as MAPK or ERK pathway. In this writing, for simplicity, the pathway will be mentioned as ERK pathway.
