# Supplementary material for: Variant‐Specific Landscape of Mutual Exclusivity Among BRAF, EGFR, and KRAS Oncogenes Reveals Overlap With Functionally Antagonistic Mutant Pairs
Source: Int J Cancer. 2026 Jun 8;159(7):1715–34. doi: 10.1002/ijc.70558 (PMC13432369; doi:10.1002/ijc.70558)
Supplement: Supplementary file 5 — Figure S1: Frequency of cancer types among the publicly available dataset queried for the current study. Figure S2: Kaplan–Meier curves for assessing Overall Survival rates in Colorectal Cancer and Non‐small Cell Lung Cancer patients' groups with BRAF, KRAS, or EGFR Mutations. Figure S3: ijc70558‐sup‐0005‐Figure S1‐S5.pdf. Figure S4: ijc70558‐sup‐0005‐Figure S1‐S5.pdf. Figure S5: Epistasis‐aware validation of variant‐level mutual exclusivity using CancerEffectSizeR. [file IJC-159-1715-s001.pdf]

Supplementary materials

Variant-Specific Landscape of Mutual Exclusivity Among BRAF, EGFR, and KRAS Oncogenes Reveals Overlap with Functionally Antagonistic Mutant Pairs

Freya Vaeyens, Jan-Patrick Hetzel, Khaldoon Abdullah, Carolien Eggermont, Catharina Olsen, Marco Mernberger, Ken Maes, Jelle Vlaeminck, Rainer Claus, Dries Vanisterbecq, Frederik Hes, Martin Pichler, Philippe Giron, Oleg Timofeev, and Maxim Noeparast

Table of Contents

Supplementary tables (separate Excel file)

Supplementary Table S1: Gene-specific variants found in human cancer samples and corresponding tissue types extracted from cBioportal and used in this study

Supplementary Table S2: Sequencing statistics of the Belgian cancer NGS dataset (based on reference genome GRCh37)

Supplementary Table S3: Classification of BRAF, KRAS, and EGFR mutations acknowledged in this study

Supplementary Table S4: Co-occurring Gene variants identified in the same cell line

Supplementary Table S5: Co-occurrence or mutual exclusivity among unilaterally class-assigned BRAF, KRAS, and EGFR gene variants found in human cancer cell lines

Supplementary Table S6: Samples with yet co-occurrence of ME scenarios with statistical significance and their related tissue types AF

Supplementary Table S7 a-f: Overall Survival data belonging to CRC and NSCLC patients with BRAF, KRAS, and EGFR mutations stratified to ME or CO groups and adjusted to five year

Supplementary Table S8: Precedent studies reveal that synthetic lethality and senescence lie behind mutual exclusivity among some oncogenic BRAF, KRAS, and EGFR events

Supplementary Table S9-11: Tables related to Figure 3

Supplementary Table S12: Gene-specific variants identified in the Belgian dataset of six tumor types

Supplementary Table S13: Cell lines used in vitro experiments

Supplementary Table S14-15: Table related to Figure S5

Supplementary figures

Supplementary Figure S1: Frequency of cancer types among the publicly available dataset queried for the current study

Supplementary Figure S2: Kaplan–Meier curves for assessing Overall Survival rates in Colorectal Cancer and Non-small Cell Lung Cancer patients' groups with BRAF, KRAS, or EGFR Mutations

Supplementary Figure S3

Supplementary Figure S4

Supplementary Figure S5: Epistasis-aware validation of variant-level mutual exclusivity using CancerEffectSizeR

Supplementary movies

Movie 1. Representative movie of PC-9 cells stably transduced with the BRAFV600E oncogene, without doxycycline (day 0-6)

Movie 2. Representative movie of PC-9 cells stably transduced with the BRAFV600E oncogene, treated with 1µg/ml doxycycline (day 0-6)

Movie 3. Representative movie of LS513 cells stably transduced with the EGFRA289V oncogene, without doxycycline (day 0-6)

Movie 4. Representative movie of LS513 cells stably transduced with the EGFRA289V oncogene, treated with 1µg/ml doxycycline (day 0-6)

Supplementary references

Supplementary tables (separate Excel file)

The supplementary tables are available in a separate Excel file, while their corresponding titles and descriptions are presented in this document.

Supplementary Table S1: Gene-specific variants found in human cancer samples and corresponding tissue types extracted from cBioPortal and used in this study

Supplementary Table S2: Sequencing statistics of the Belgian cancer NGS dataset (based on reference genome GRCh37)

Supplementary Table S3: Classification of BRAF, KRAS, and EGFR mutations acknowledged in this study

According to a widely accepted classification (1), BRAF mutations are classified into three classes. Class I variants, which include the typical BRAFV600E, are known to function as constitutively active monomers and can signal independent of upstream RAS or another RAF isoform CRAF. Atypical BRAF mutations belong to either class II or class III. Class II is suggested

to activate the ERK pathway independent of RAS and CRAF but signals as homodimers and activates the ERK pathway at lower levels than class I. BRAFG469A is the well-studied class II BRAF in cancer, which activates ERK slightly less than BRAFV600E 2. Class III comprises the kinase-impaired BRAFmutants, which activate the ERK pathway via allosteric transactivation of the CRAF in a RAS-dependent manner (2,3).

For KRAS mutations, we considered the most updated classification described by Johnson et al. (4). Concerning cancer-related KRAS mutations, three classes are defined according to the impact of mutations on two critical features of the KRAS protein. KRAS can hydrolyze GTP and oscillate back to inactive conformation (4). Mutations that lead to loss of GTP-hydrolyzing function are classified as class I (Hydrolysis) (4) Moreover, Guanine Nucleotide Exchange factors (GEFs) and corresponding scaffolding proteins enable KRAS to dislodge GDP, accept GTP, and transit to the active conformation (4). Mutations that lead to the gain of the KRAS Exchange function are classified as class II (Exchange). Furthermore, the third class comprises the KRAS mutations that affect both the Hydrolysis and Exchange (class III: Hybrid) (4). Hydrolysis KRAS mutations are the most prevalent KRAS variants in human cancer (4). With regards to EGFR mutations, we considered a recent classification described by Robichaux et.al (5) which is based on the structural impact of mutations on EGFR protein and, in particular, EGFR's drug-binding pocket (DBP) as well as the consequences of mutations on drug response. Accordingly, EGFR mutations are classified into four groups. Classical-likes are those mutations positioned relatively far from DBP and have no or inconsiderable effect on EGFR affinity for the three generations of the Tyrosine Kinase Inhibitors (TKIs) (5). T790M- like class includes those EGFR variants with at least one mutation in the hydrophobic core of the EGFR ATP binding pocket, at the gatekeeper residue alone or with other mutations (5). T790M-like mutations increase the affinity of EGFR for ATP and present two sub-groups that we distinguished in our analyses, namely T790M-like-3R and T790M-like-3S; the latter can be sensitive to 3rd generation TKIs. Exon 20 loop insertions (Ex20ins-L) involve the C- terminal loop of the αC-helix and induce a global impact on EGFR DBP by altering both P-loop (inward surface of the ATP binding pocket) and αC-helix conformations. They indirectly affect the affinity for inhibitors. These mutations exhibit differential sensitivity to TKIs (5). The P-loop αC-helix compressing (PACC) represents mutations close to DBP. Their conformational impact, less than Ex20ins variants, affects either the P-loop or αC-helix alone or concomitantly (5). They directly or indirectly (like Ex20ins-L) influence the drug binding. These mutations might sensitize to 2nd generation TKIs. Note that our dataset was minimal (3) with Ex20ins and PACC variants. Therefore, we did not distinguish corresponding sub-groups (for detailed and more comprehensive descriptions of EGFR classes, refer to Robichaux et.al. (5).

Supplementary Table S4: Co-occurring Gene variants identified in the same cell line

The only CO BRAF variants predicted to be drivers were BRAFG464E/V, belonging to class II. On the other hand, most of the KRAS mutations in this CO group belonged to the Hydrolysis class, and only one belonged to the Exchange class. Among EGFR∩KRAS cell lines, no classified EGFR mutation existed. EGFRA864V, EGFRV292M, and EGFRA289V were the only variants predicted to be the driver. KRAS mutations in EGFR∩KRAS were more diverse than in BRAF∩KRAS, encompassing all three classes, but still dominated by Hydrolysis variants. Among BRAF∩EGFR cell lines, BRAFV600E co-occurred only with non-driver/non-classified EGFR variants.

Supplementary Table S5: Co-occurrence or mutual exclusivity among unilaterally class-assigned BRAF, KRAS, and EGFR gene variants found in human cancer cell lines

The same analyses as in Table 1, but on 1570 mutant cell line data, available in Cancer Cell Line Encyclopedia (Broad, 2019), and considering the ones with BRAF, KRAS, or EGFR mutations (see Figure 1A). When ORs do not follow the same trend as in patients' samples, they are mentioned in red. Class I and III BRAFs were absent in BRAF∩KRAS cell lines.

Supplementary Table S6: Samples with yet co-occurrence of ME scenarios with statistical significance and their related tissue types

AF: Allele Frequency in the tumor, **na**: not available. AFs <0.1 are mentioned in red. Note that non-small lung cancer and colorectal cancer account for the 2nd and 3rd most frequent samples in our queried dataset after breast cancer (see Supplementary Figure 1). These two cancer types are known to recurrently harbor KRAS, BRAF, or EGFR mutations. We calculated binomial probabilities of encountering exactly these cancer types upon random sampling. The likelihood of BRAF∩EGFR occurring exactly 1 out of 1 time in Non-Small Cell Lung Cancer was calculated as p = 0.1010, CI = (0.03, 1.00), the same for the following was inferred: KRAS∩BRAF 5 of 6 of Colorectal Cancer p = 0.0000, CI = (0.36, 1.00), KRAS∩BRAF 1 of 6 of Non-Small Cell Lung Cancer p = 0.4721, CI = (0.00, 0.64) and the KRAS∩EGFR 2 of 2 of Non-Small Cell Lung Cancer p = 0.0102, CI = (0.16, 1.00). Note that confidence intervals for these p-values are so large, hindering any robust statistical conclusions.

Supplementary Table S7 a-f: Overall Survival data belonging to CRC and NSCLC patients with BRAF, KRAS, and EGFR mutations stratified to ME or CO groups and adjusted to five year

Supplementary Table S8: Precedent studies reveal that synthetic lethality and senescence lie behind mutual exclusivity among some oncogenic BRAF, KRAS, and EGFR events

For each **study**, the investigated **Gene** variants and the study **Model** where the **Observed phenotype** was investigated are summarized. In a subsequent study, Unni et al. Discovered DUSP6 as a signaling node that governs the observed phenotype (6).

Supplementary Table S9-11: Tables related to Figure 3

Supplementary Table S12: Gene-specific variants identified in the Belgian dataset of six tumor types

Supplementary Table S13: Cell lines used in vitro experiments

Supplementary Table S14-15: Table related to Figure S5

Supplementary figures

Supplementary Figure S1: Frequency of cancer types among the publicly available dataset queried for the current study

Supplementary Figure S2: Kaplan–Meier curves for assessing Overall Survival rates in Colorectal Cancer and Non-small Cell Lung Cancer patients' groups with BRAF, KRAS, or EGFR Mutations

Overall survival rates were pairwise compared among corresponding ME and CO groups as described in the methodology. In (**A-C**) OS of CRC patients' groups and in (**D-F**), the same for different NSCLC patients' groups are displayed. (**A**) Pairwise comparison of OS among KRAS mutant patients (n=1032) vs. BRAF mutant patients (n=273), which yields the p-value=0.011, KRAS mutant patients vs. BRAF∩KRAS mutant patients (n=31) yields the p-value=0.122, and BRAF vs. BRAF∩KRAS mutant patients yields the p-value=0.717. (**B**) The same pairwise comparison is displayed for EGFR mutant patients (n=39) vs. KRAS mutant patients (n=1040), which yields the p-value=0.456, EGFR mutant patients vs.EGFR∩KRAS mutant patients (n=23): the p-value=0.150, and KRAS mutant patients vs.EGFR∩KRAS mutant patients: the p-value=0.119. (**C**) The same comparison is displayed for EGFR mutant patients (n=43) vs. BRAF mutant patients (n=285), which yields p-value=0.753, EGFR mutant patients vs. EGFR∩BRAF mutant patients (n=19): p-value=0.382, and BRAF mutant patients vs. EGFR∩BRAF mutant patients: p-value=0.178. (**D**) Pairwise comparison of OS among KRAS mutant patients (n=1183) vs.BRAF mutant patients (n=235), which yields the p-value=0.269, KRAS mutant patients vs. BRAF∩KRAS mutant patients (n=30) yields the p-value=0.265, and BRAF mutant patients vs. BRAF∩KRAS mutant patients: the p-value=0.527. (**E**) The same is displayed for EGFR mutant patients (n=1203) vs. KRAS mutant patients (n=1178), which yields the p-value<0.001, EGFR mutant patients vs. EGFR∩KRAS mutant patients (n=35) yields the p-value<0.001, and KRAS mutant patients vs. EGFR∩KRAS mutant patients yields the p-value=0.057. (**F**) The same comparison is displayed for EGFR mutant patients (n=1204) vs.BRAF mutant patients (n=231), which yields p-value=0.282, EGFR mutant patients vs. EGFR∩BRAF mutant patients (n=34): the p-value=0.253, and BRAF mutant patients vs.EGFR∩BRAF mutant patients: the p-value=0.560.

Supplementary Figure S3

(**A**) Detection of GFP expression by flow cytometry. Cells were treated for 48h with 1µg/ml doxycycline to induce expression of the transgenes, detached with 0.05% trypsin (Gibco), and collected in cell culture medium. After 5 min centrifugation at 500 g, supernatants were aspirated, cell pellets were resuspended in PBS, and measured using an LSRII flow cytometer (BD Biosciences). (**B**) PCR detection of stably integrated transgenes, performed using primer pairs specific for the BRAF or EGFR cDNA present in the lentiviral constructs, with water as NTC (negative control). (**C**) Sanger sequencing of PCR fragments from (**B**), confirming the presence of the wild-type or mutant cDNA transgene. (**D**) RT-qPCR analysis of GFP and BRAF or EGFR expression in stably transduced cell lines before and after induction. Results are shown relative to housekeeping gene expression (GAPDH), calculated as $2^{(-\Delta Ct)}$. In one of

two independent experiments, the average values and standard deviations of three technical replicates are shown.

Supplementary Figure S4

(**A**) Representative images from the live video imaging, taken at indicated time-points from induced (doxycycline-treated) and non-treated PC-9 cells. (**B**) Representative images from the live video imaging, taken at indicated time-points from induced (doxycycline-treated) and non-treated LS513 cells.

Supplementary Figure S5: Epistasis-aware validation of variant-level mutual exclusivity using CancerEffectSizeR

Comparative analysis of variant-pair mutual exclusivity in the PCAWG pan-cancer whole-genome cohort (2,922 tumors; 413 with mutations in KRAS, EGFR, or BRAF). (**A**) Concordance between p-values derived from an epistasis-aware CancerEffectSizeR analysis and a Fisher's exact test–based pipeline for all evaluable variant pairs, demonstrating strong overall agreement. (**B**) CancerEffectSizeR p-values for variant pairs previously identified as significantly mutually exclusive in the full cohort; the most significant association, KRAS G12D–BRAFV600E, is consistently recovered. Reduced concordance for other pairs reflects limited statistical power and variant representation in the smaller PCAWG dataset. Somatic mutation data were obtained from the Pan-Cancer Analysis of Whole Genomes study via cBioPortal.

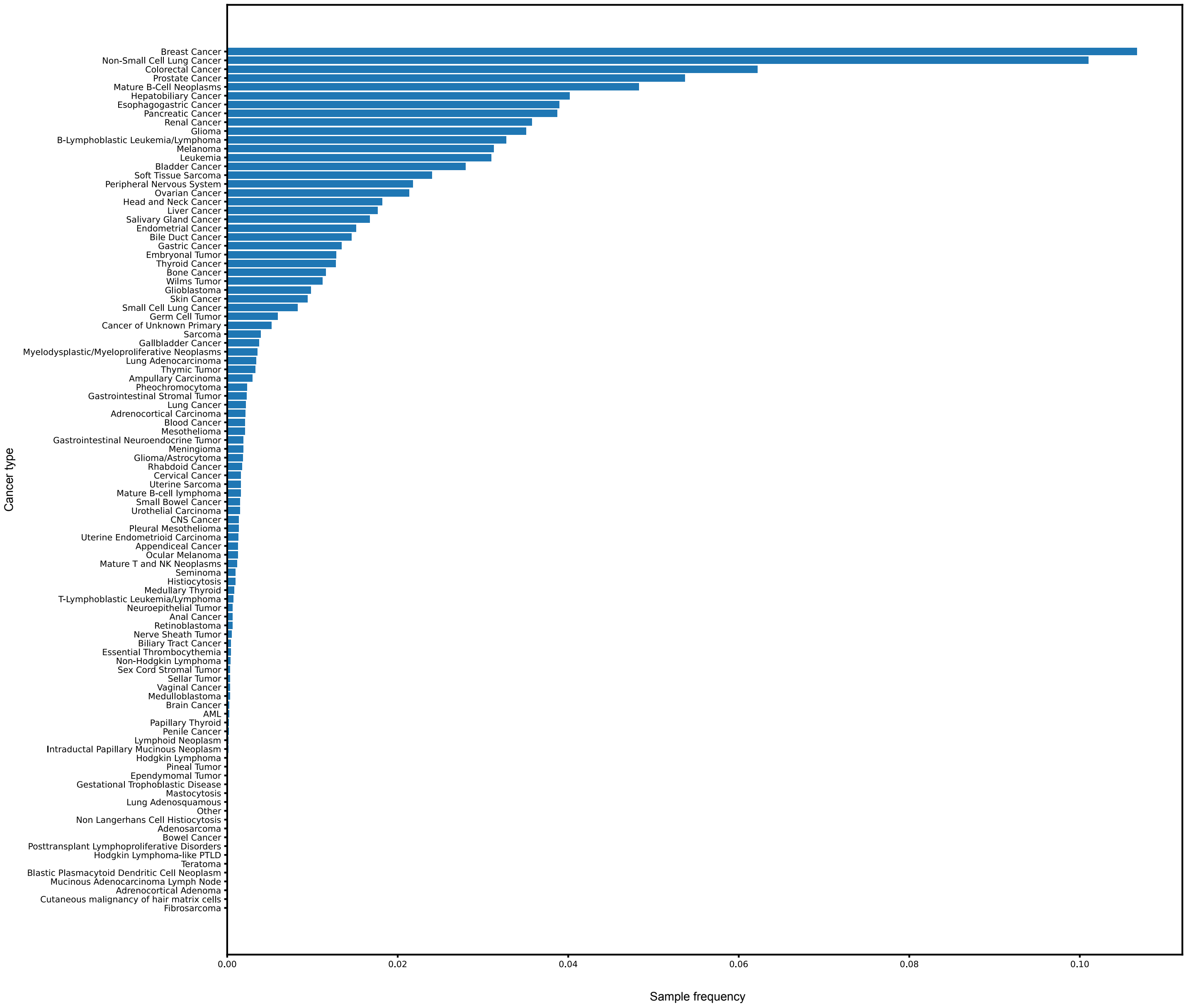
Cancer type
Sample frequency
0.00
0.02
0.04
0.06
0.08
0.10
Breast Cancer
Non-Small Cell Lung Cancer
Colorectal Cancer
Prostate Cancer
Mature B-Cell Neoplasms
Hepatobiliary Cancer
Esophagogastric Cancer
Pancreatic Cancer
Renal Cancer
Glioma
B-Lymphoblastic Leukemia/Lymphoma
Melanoma
Leukemia
Bladder Cancer
Soft Tissue Sarcoma
Peripheral Nervous System
Ovarian Cancer
Head and Neck Cancer
Liver Cancer
Salivary Gland Cancer
Endometrial Cancer
Bile Duct Cancer
Gastric Cancer
Embryonal Tumor
Thyroid Cancer
Bone Cancer
Wilms Tumor
Glioblastoma
Skin Cancer
Small Cell Lung Cancer
Germ Cell Tumor
Cancer of Unknown Primary
Sarcoma
Gallbladder Cancer
Myelodysplastic/Myeloproliferative Neoplasms
Lung Adenocarcinoma
Thymic Tumor
Ampullary Carcinoma
Pheochromocytoma
Gastrointestinal Stromal Tumor
Lung Cancer
Adrenocortical Carcinoma
Blood Cancer
Mesothelioma
Gastrointestinal Neuroendocrine Tumor
Meningioma
Glioma/Astrocytoma
Rhabdoid Cancer
Cervical Cancer
Uterine Sarcoma
Mature B-cell lymphoma
Small Bowel Cancer
Urothelial Carcinoma
CNS Cancer
Pleural Mesothelioma
Uterine Endometrioid Carcinoma
Appendiceal Cancer
Ocular Melanoma
Mature T and NK Neoplasms
Seminoma
Histiocytosis
Medullary Thyroid
T-Lymphoblastic Leukemia/Lymphoma
Neuroepithelial Tumor
Anal Cancer
Retinoblastoma
Nerve Sheath Tumor
Biliary Tract Cancer
Essential Thrombocythemia
Non-Hodgkin Lymphoma
Sex Cord Stromal Tumor
Sellar Tumor
Vaginal Cancer
Medulloblastoma
Brain Cancer
AML
Papillary Thyroid
Penile Cancer
Lymphoid Neoplasm
Intraductal Papillary Mucinous Neoplasm
Hodgkin Lymphoma
Pineal Tumor
Ependymomal Tumor
Gestational Trophoblastic Disease
Mastocytosis
Lung Adenosquamous
Other
Non Langerhans Cell Histiocytosis
Adenosarcoma
Bowel Cancer
Posttransplant Lymphoproliferative Disorders
Hodgkin Lymphoma-like PTLD
Teratoma
Blastic Plasmacytoid Dendritic Cell Neoplasm
Mucinous Adenocarcinoma Lymph Node
Adrenocortical Adenoma
Cutaneous malignancy of hair matrix cells
Fibrosarcoma

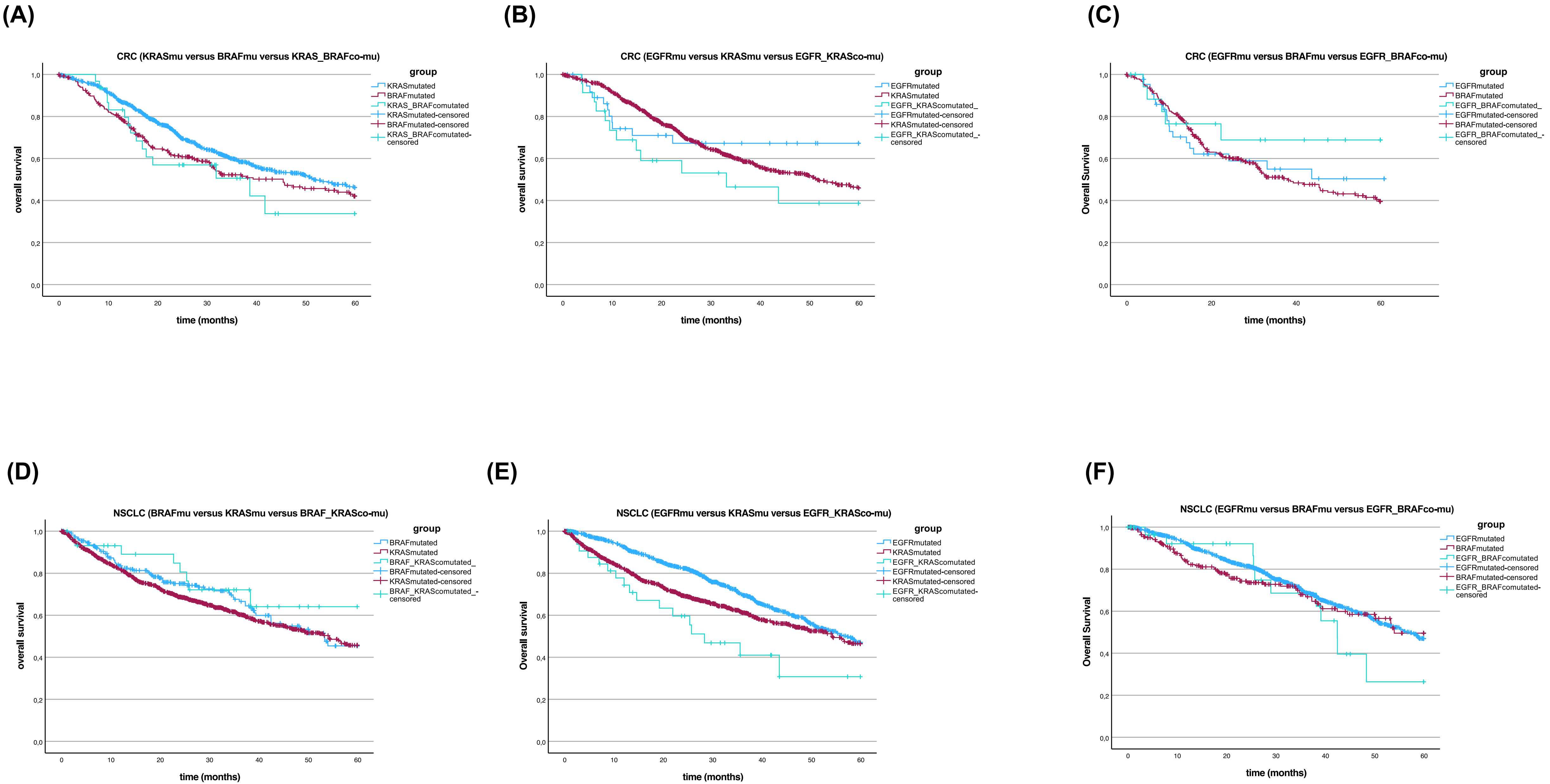

(A)
CRC (KRASmu versus BRAFmu versus KRAS_BRAFco-mu)
overall survival
time (months)
group
KRASmutated
BRAFmutated
KRAS_BRAFcomutated
KRASmutated-censored
BRAFmutated-censored
KRAS_BRAFcomutated-censored
(B)
CRC (EGFRmu versus KRASmu versus EGFR_KRASco-mu)
overall survival
time (months)
group
EGFRmutated
KRASmutated
EGFR_KRAScomutated_
EGFRmutated-censored
KRASmutated-censored
EGFR_KRAScomutated_-censored
(C)
CRC (EGFRmu versus BRAFmu versus EGFR_BRAFco-mu)
Overall Survival
time (months)
group
EGFRmutated
BRAFmutated
EGFR_BRAFcomutated_
EGFRmutated-censored
BRAFmutated-censored
EGFR_BRAFcomutated_-censored
(D)
NSCLC (BRAFmu versus KRASmu versus BRAF_KRASco-mu)
overall survival
time (months)
group
BRAFmutated
KRASmutated
BRAF_KRAScomutated_
BRAFmutated-censored
KRASmutated-censored
BRAF_KRAScomutated_-censored
(E)
NSCLC (EGFRmu versus KRASmu versus EGFR_KRASco-mu)
Overall survival
time (months)
group
EGFRmutated
KRASmutated
EGFR_KRAScomutated
EGFRmutated-censored
KRASmutated-censored
EGFR_KRAScomutated-censored
(F)
NSCLC (EGFRmu versus BRAFmu versus EGFR_BRAFco-mu)
Overall Survival
time (months)
group
EGFRmutated
BRAFmutated
EGFR_BRAFcomutated
EGFRmutated-censored
BRAFmutated-censored
EGFR_BRAFcomutated-censored

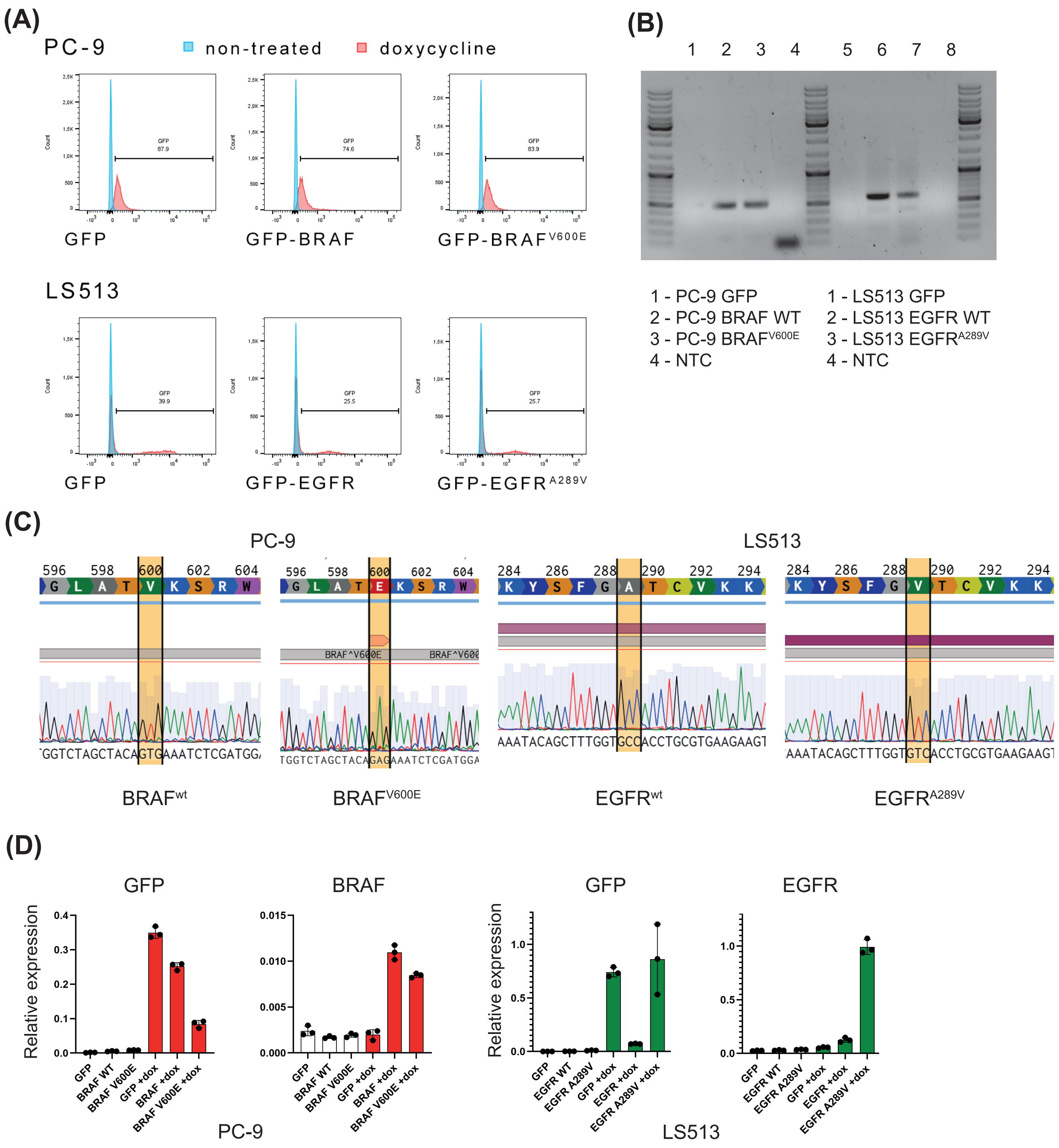

(A)
PC-9
non-treated
doxycycline
GFP
GFP-BRAF
GFP-BRAF V600E
LS513
GFP
GFP-EGFR
GFP-EGFR A289V
(B)
1 2 3 4 5 6 7 8
1 - PC-9 GFP
2 - PC-9 BRAF WT
3 - PC-9 BRAF V600E
4 - NTC
1 - LS513 GFP
2 - LS513 EGFR WT
3 - LS513 EGFR A289V
4 - NTC
(C)
PC-9
LS513
GGTCTAGCTACAGTGAAATCTCGATGG
TGGTCTAGCTACAGAGAAATCTCGATGGA
AAATACAGCTTTGGTGCCACCTGCGTGAAGAAGT
AAATACAGCTTTGGTGTCACCTGCGTGAAGAAGT
BRAFwt
BRAF V600E
EGFRwt
EGFR A289V
(D)
GFP
BRAF
GFP
EGFR
Relative expression
GFP
BRAF WT
BRAF V600E
GFP +dox
BRAF +dox
BRAF V600E +dox
PC-9
GFP
EGFR WT
EGFR A289V
GFP +dox
EGFR +dox
EGFR A289V +dox
LS513

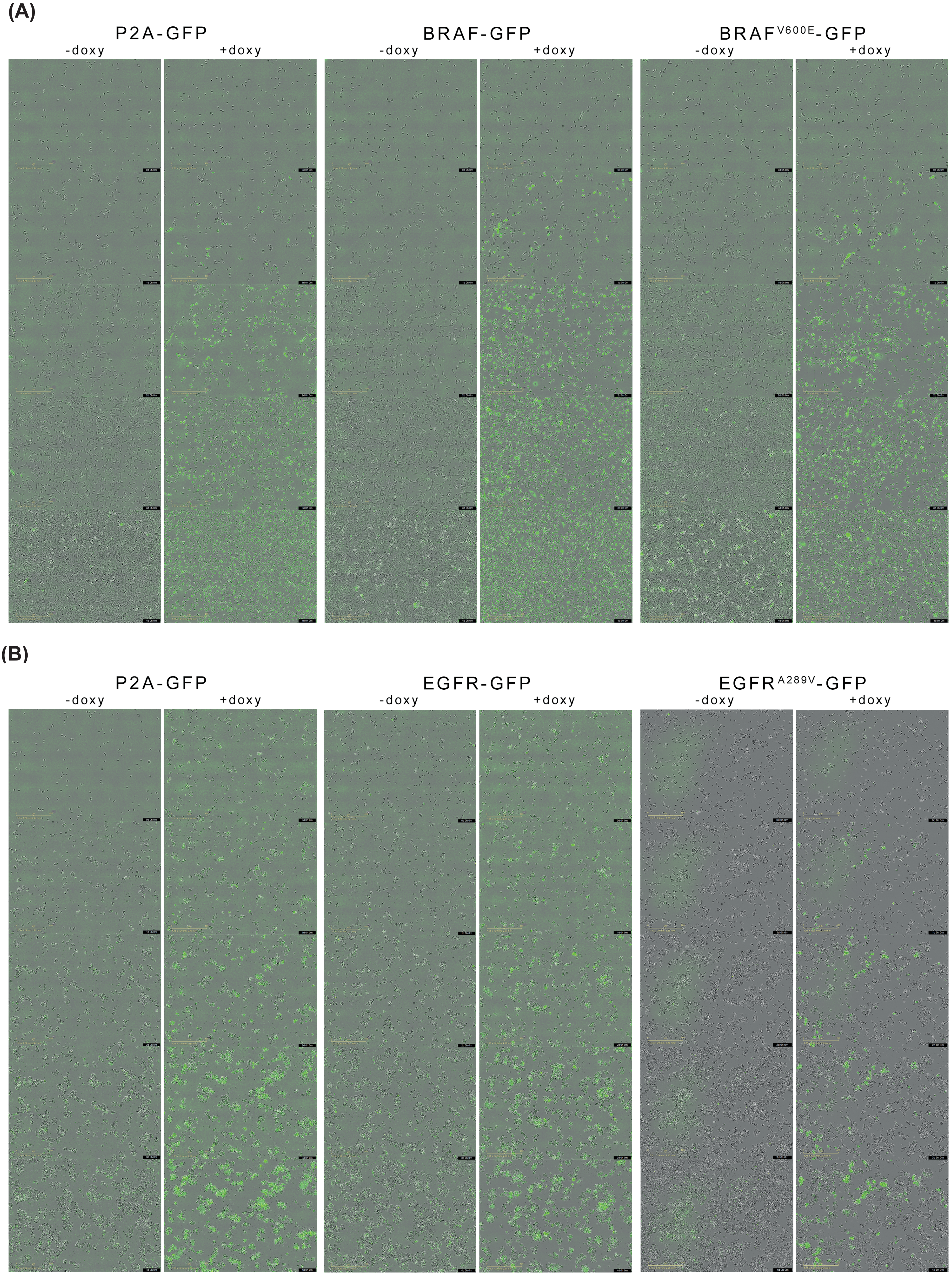
(A)
P2A-GFP
-doxy
+doxy
BRAF-GFP
-doxy
+doxy
BRAF^{V600E}-GFP
-doxy
+doxy
(B)
P2A-GFP
-doxy
+doxy
EGFR-GFP
-doxy
+doxy
EGFR^{A289V}-GFP
-doxy
+doxy

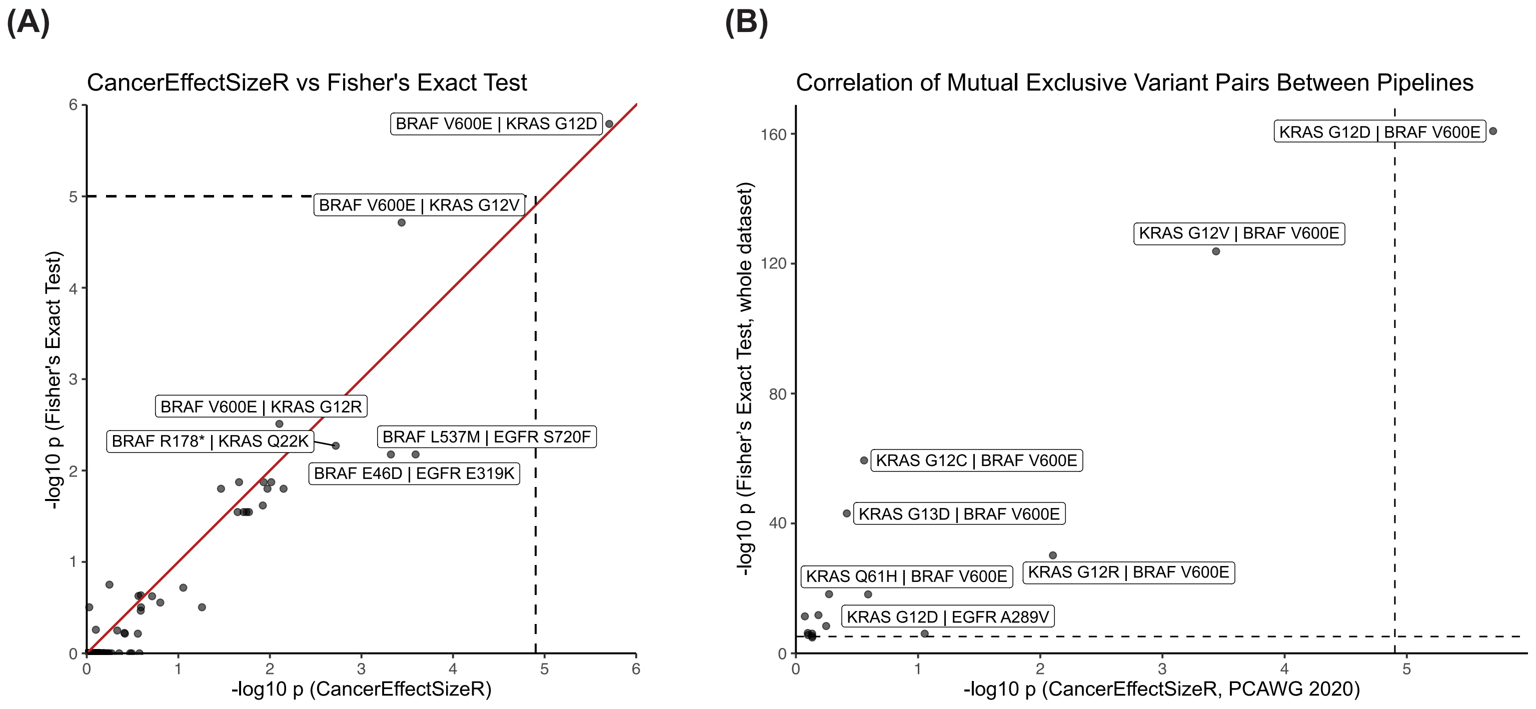
(A)
CancerEffectSizeR vs Fisher's Exact Test
BRAF V600E | KRAS G12D
BRAF V600E | KRAS G12V
BRAF V600E | KRAS G12R
BRAF R178* | KRAS Q22K
BRAF L537M | EGFR S720F
BRAF E46D | EGFR E319K
-log10 p (Fisher's Exact Test)
-log10 p (CancerEffectSizeR)
(B)
Correlation of Mutual Exclusive Variant Pairs Between Pipelines
KRAS G12D | BRAF V600E
KRAS G12V | BRAF V600E
KRAS G12C | BRAF V600E
KRAS G13D | BRAF V600E
KRAS Q61H | BRAF V600E
KRAS G12R | BRAF V600E
KRAS G12D | EGFR A289V
-log10 p (Fisher's Exact Test, whole dataset)
-log10 p (CancerEffectSizeR, PCAWG 2020)

Supplementary movies

Movie 1. Representative movie of PC-9 cells stably transduced with the BRAFV600E oncogene, without doxycycline (day 0-6).

Movie 2. Representative movie of PC-9 cells stably transduced with the BRAFV600E oncogene, treated with 1µg/ml doxycycline (day 0-6).

Movie 3. Representative movie of LS513 cells stably transduced with the EGFRA289V oncogene, without doxycycline (day 0-6).

Movie 4. Representative movie of LS513 cells stably transduced with the EGFRA289V oncogene, treated with 1µg/ml doxycycline (day 0-6).

Supplementary references

1. Dankner, M., Rose, A. A. N., Rajkumar, S., Siegel, P. M. & Watson, I. R. Classifying BRAF alterations in cancer: New rational therapeutic strategies for actionable mutations. Oncogene 37, 3183–3199 (2018).

2. Noeparast, A. et al. Non-V600 BRAF mutations recurrently found in lung cancer predict sensitivity to the combination of Trametinib and Dabrafenib. Oncotarget 8, 60094–60108 (2017).

3. Yao, Z. et al. BRAF Mutants Evade ERK-Dependent Feedback by Different Mechanisms that Determine Their Sensitivity to Pharmacologic Inhibition. Cancer Cell 28, 370–383 (2015).

4. Johnson, C., Burkhart, D. L. & Haigis, K. M. Classification of KRAS-Activating Mutations and the Implications for Therapeutic Intervention. Cancer Discov. 12, 913–923 (2022).

5. Robichaux, J. P. et al. Structure-based classification predicts drug response in EGFR-mutant NSCLC. Nature 597, 732–737 (2021).

6. Unni, A. M. et al. Hyperactivation of ERK by multiple mechanisms is toxic to RTK-RAS mutation-driven lung adenocarcinoma cells. Elife 7, 1–24 (2018).
